# Ammoniating Covalent Organic Framework (COF) for High‐Performance and Selective Extraction of Toxic and Radioactive Uranium Ions

**DOI:** 10.1002/advs.201900547

**Published:** 2019-06-27

**Authors:** Xiao Hong Xiong, Zhi Wu Yu, Le Le Gong, Yuan Tao, Zhi Gao, Li Wang, Wen Hui Yin, Li Xiao Yang, Feng Luo

**Affiliations:** ^1^ State Key Laboratory of Nuclear Resources and Environment School of Chemistry, Biology and Materials Science East China University of Technology Nanchang 330013 P. R. China; ^2^ High Magnetic Field Laboratory Chinese Academy of Sciences Hefei 230031 Anhui P. R. China

**Keywords:** covalent organic frameworks (COFs), coordination interactions, ion exchange, synergic effect, uranium adsorption

## Abstract

An ideal porous adsorbent toward uranium with not only large adsorption capacity and high selectivity but also broad applicability even under rigorous conditions is highly desirable but still extremely scarce. In this work, a porous adsorbent, namely [NH_4_]^+^[COF‐SO_3_^−^], prepared by ammoniating a SO_3_H‐decorated covalent organic framework (COF) enables remarkable performance for uranium extraction. Relative to the pristine SO_3_H‐decorated COF (COF‐SO_3_H) with uranium adsorption capacity of 360 mg g^−1^, the ammoniated counterpart of [NH_4_]^+^[COF‐SO_3_^−^] affords ultrahigh uranium uptake up to 851 mg g^−1^, creating a 2.4‐fold enhancement. Such a value is the highest among all reported porous adsorbents for uranium. Most importantly, a large distribution coefficient, *K*_d_^U^, up to 9.8 × 10^6^ mL g^−1^ is observed, implying extremely strong affinity toward uranium. Consequently, [NH_4_]^+^[COF‐SO_3_^−^] affords highly selective adsorption of uranium over a broad range of metal ions such as S_U/Cs_ = 821, S_U/Na_ = 277, and S_U/Sr_ = 124, making it as effective uranium adsorbent from seawater, resulting in amazing uranium adsorption capacity of 17.8 mg g^−1^. Moreover, its excellent chemostability also make it an effective uranium adsorbent even under rigorous conditions (pH = 1, 8, and 3 m acidity).

Nuclear energy is widely considered as one of the clean energy sources. However, the increasing demand in nuclear energy inevitably leads to some serious troubles in the aspect of environment and health from the retirement of nuclear power plants, the abundant residual solution of mining, nuclear waste solution, and even urgent nuclear events.^1^ This urgently needs advanced materials with great chemostability (especially in rigorous conditions such as strong acidic surrounding) and strong radiation resistance to effectively remove and recover uranium.^2^ On the other hand, it is also imminently concerned that the shortage of uranium storage content in earth, in terms of the present state of the art, could not meet the sustainable development. Accordingly, seawater contains about 4.5 billion tons of uranium, suggesting its promising potential as uranium source. But its extremely low uranium concentration and contrarily huge content of other interference metal ions (such as Na^+^, K^+^, and Sr^+^) makes the selective extraction of uranium from seawater a challenging task.^3^

Generally speaking, to effectively remove or recover uranium from nuclear waste solution or seawater, the ideal porous adsorbent must obey four important criterions, viz. large adsorption capacity, high selectivity, rapid kinetics, and good recycle use.^2^, ^3^ It is disclosed that the traditional porous materials such as clays, activated carbons, and zeolites often show low adsorption capacity, weak selectivity, and slow kinetics.^2^, ^3^ To this end, some advanced materials based on the ion exchange mechanism is shown to be effective. For example, KMS‐1,^4^ KTS‐3,^5^ FJSM‐SnS,^6^ and FJSM‐GAS‐2^7^ show high U adsorption capacity of 380, 347, 338, and 196 mg g^−1^, respectively. Fast adsorption kinetics derived from the inherent nature of ion exchange was also observed among these reported metal chalcogenides.^4^, ^5^, ^6^, ^7^

Alternatively, we can anchor various functionalized organic units within the pore wall of metal–organic frameworks (MOFs),^8^ a new porous platform built on metal ions and organic ligands, to give coordination interaction for selective capture of uranium. For instance, the MOF with free‐standing carboxyl groups shows uranyl uptake of 115 mg g^−1^,[qv: 8e] while the phosphorylurea‐ and diethylenetriamine‐anchored MOF affords uranyl uptake of 217 and 350 mg g^−1^,[qv: 8a] respectively. However the long‐term stability of MOFs under acidic or alkaline condition still remains challenging.^9^

By contrast, another promising porous platform of covalent organic framework (COF) not only shows high chemostability but also can be facilely anchored with functionalized organic units by the postsynthetic modification,^10^ as well as their compose just contains light elements, making COF as a promising porous uranium adsorbent.^11^ For example, Ma and coworkers showed that the amidoxime‐functionalized COFs enable remarkable uranyl adsorption performance up to 530 mg g^−1^.[qv: 11a]

Despite these pioneered outstanding achievements, however, to the best of our knowledge, the porous adsorbents showing effective uranium adsorption ability under both 3 m acidic and weakly alkaline environment (pH = 8), which to some extent reflects the real‐world environment of nuclear waste solution and seawater, are still highly rare.^11^, ^12^ This is mainly due to, a) difficulties in synthesis of robust porous adsorbent that can survive under both 3 m acidity and pH = 8, and b) powerful competition that requires the porous adsorbent with strong affinity toward uranium ions, thus overcoming the serious effects from acid or CO_3_^2−^. In this regard, the recent developed COF material could be a right candidate due to its high chemostability and structural designability. Accordingly, anchoring acidic organic unit such as ‐SO_3_H within the COF scaffold is theoretically expected to enhance its acid‐ and alkaline‐ resistant ability, while the ‐SO_3_H unit could enable high affinity toward uranyl via coordination interaction,^13^ thus predicting high performance in uranium adsorption.

Taking the above discussion into account, in this work we focused on the exploration of a combined approach composed of ion exchange and coordination interaction through anchoring the pore wall with functionalized organic unit, expecting to target a synergistic effect and consequently to generate superior material with superperformance. To this end, as shown in **Scheme** 1, we first deliberately prepared the SO_3_H‐anchored COF (COF‐SO_3_H). Then the ion‐exchange material of [NH_4_]^+^[COF‐SO_3_^−^] was obtained by immerging COF‐SO_3_H in NH_3_·H_2_O. Most interestingly, such material also contains abundant ‐SO_3_^−^ unit in the pore wall that could implement the coordination interaction toward uranyl. As expected, superperformance is observed for [NH_4_]^+^[COF‐SO_3_^−^], giving a recorded adsorption capacity up to 851 mg g^−1^, strong affinity with *K*_d_^U^ up to 9.8 × 10^6^ mL g^−1^, big selectivity over a broad range of metal ions (e.g., S_U/Cs_ = 821), general applicability even in both 3 m acidity and pH = 8, large uranium recovery power up to 17.8 mg g^−1^ from seawater, and excellent chemostability for recycle use.

**Scheme 1:**
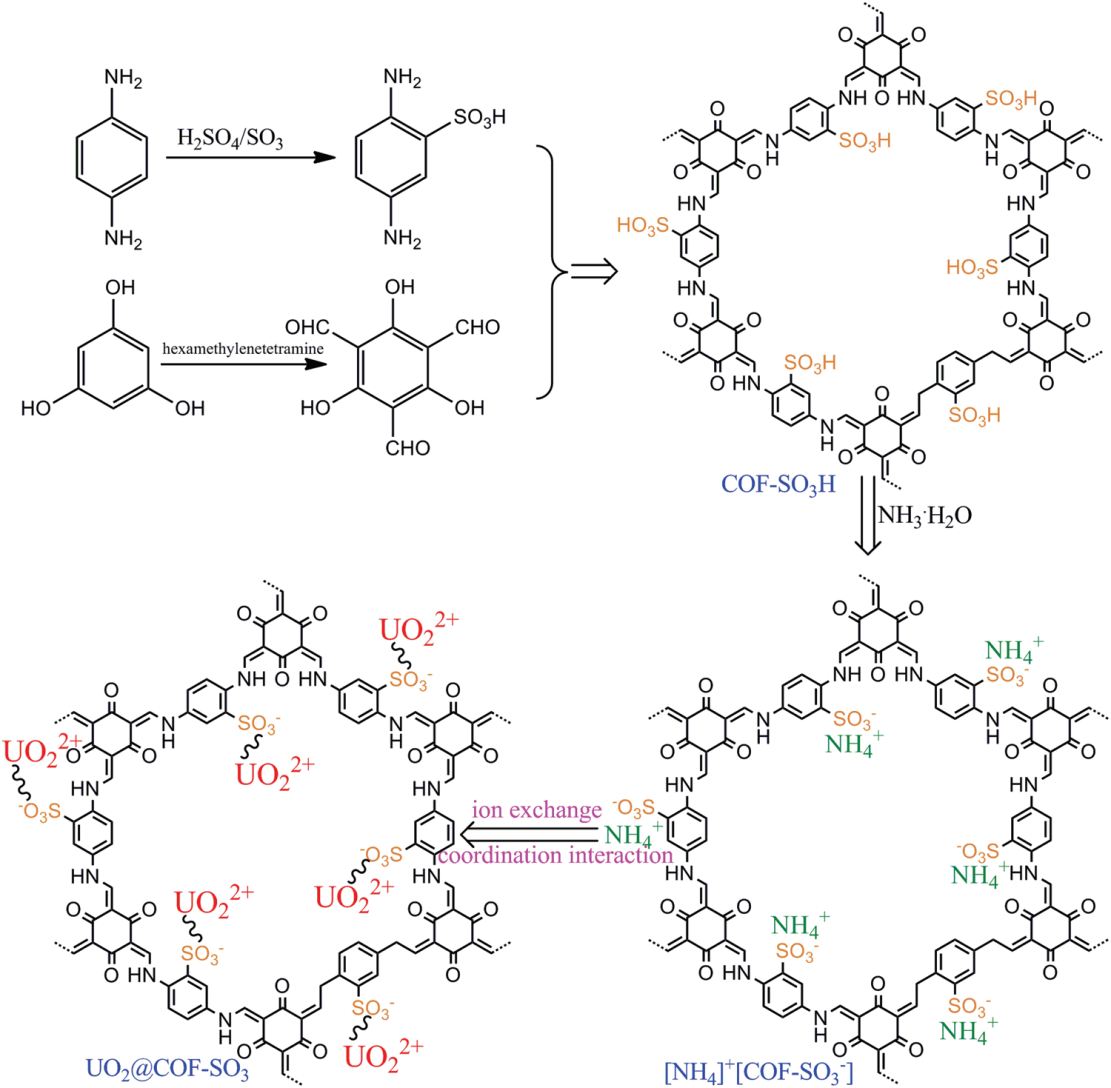
Synthetic scheme of SO_3_H‐decorated COF (COF‐SO_3_H) and the ammoniated material of [NH_4_]^+^[COF‐SO_3_^−^]. A proposal of combined approach involved in both ion exchange and coordination interaction.

The COF‐SO_3_H samples were prepared by the solvothermal method. [NH_4_]^+^[COF‐SO_3_^−^] was obtained by ammoniating COF‐SO_3_H in NH_3_·H_2_O. The synthetic detail is listed in the Supporting Information. The crystalline nature and structures of COF‐SO_3_H and [NH_4_]^+^[COF‐SO_3_^−^] were determined by powder X‐ray diffraction (PXRD) and the Materials Studio Forcite molecular dynamics module method. It is clear that the experimental PXRD patterns match well with the simulated data (Figure S1, Supporting Information). As shown in **Figure** 1, the COF‐SO_3_H shows the eclipsed stacking ABA mode, where ‐SO_3_H units among adjacent layers hold the opposite arrangement fashion rather than the common same‐side mode (Figure 1a,b). Deprotonation is observed in [NH_4_]^+^[COF‐SO_3_^−^], and NH_4_^+^ ions are located at the pore and further stabilized by hydrogen bonds (Figure 1c).

**Figure 1:**
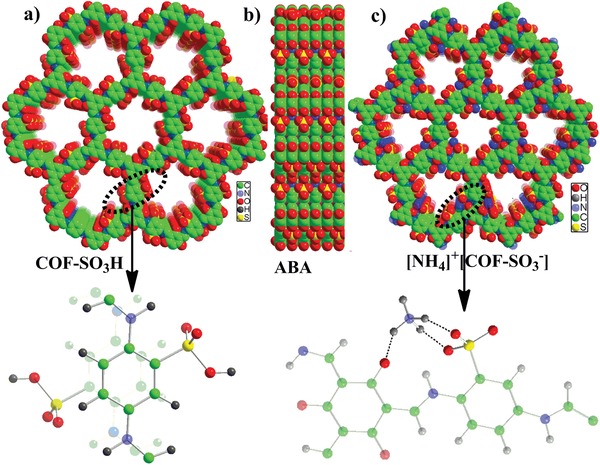
Space‐filling view of the structure of a,b) COF‐SO_3_H and c) [NH_4_]^+^[COF‐SO_3_^−^]. The COF‐SO_3_H shows the ABA stacking fashion, which means that the arrangement of ‐SO_3_H in the A and B layer is in an opposite mode rather than the common same‐side mode (see the insert). The location of NH_4_^+^ and the hydrogen‐bond interactions between NH_4_^+^ and COF skeleton are highlighted (see the insert).

To further characterize the as‐synthesized COF‐SO_3_H and [NH_4_]^+^[COF‐SO_3_^−^] materials, infrared spectrum (IR), scanning electron microscopy (SEM), energy dispersive spectrometry (EDS), X‐ray photoelectron spectroscopy (XPS), and ^13^C cross polarization magic angle spinning (CPMAS) solid‐state NMR spectroscopy were carried out. **Figure** 2a discloses the nanofiber morphology of COF‐SO_3_H, while similar morphology was maintained in [NH_4_]^+^[COF‐SO_3_^−^] (Figure 2b). In the elemental distribution mapping of EDS, the C, N, O, S elements are uniformly distributed in both samples, and as expected, [NH_4_]^+^[COF‐SO_3_^−^] displays enhanced N content (Figure 2c,d). As observed in the literature, similar trend of IR bonds at ≈1272 m^−1^ (—C—N) and ≈1586 cm^−1^(—C=C) strongly suggests the formation of β‐ketoenamine‐based framework structure (Figure S2, Supporting Information).^14^ The existence of ‐SO_3_H unit in COF‐SO_3_H is confirmed by the typical IR bonds at 1079, 814, 703, and 533 cm^−1^, while the deprotonation and existence of NH_4_^+^ in [NH_4_]^+^[COF‐SO_3_^−^] is attested by the disappearance at 725 cm^−1^ (S—O—H) and formation of new peaks at 3155 cm^−1^ (NH_4_^+^ with hydrogen bonds), relative to the pristine COF‐SO_3_H samples (Figure S2, Supporting Information). The structure is further surveyed by XPS analysis. The complete survey XPS plots of COF‐SO_3_H and [NH_4_]^+^[COF‐SO_3_^−^] are shown in Figure S3 in the Supporting Information, where C, O, N, and S elements are observed in the spectra. In the high‐resolution spectra of C1s, the peaks belonging to C=C, C—C, C—N, C=O, and C—S is rationally isolated, further confirming the β‐ketoenamine‐based framework structure in COF‐SO_3_H and [NH_4_]^+^[COF‐SO_3_^−^]. Additional evidence to support the β‐ketoenamine‐based framework structure is the observation of strong signal at 400 eV in the high‐resolution N1s spectra (Figure S4, Supporting Information), for COF‐SO_3_H and [NH_4_]^+^[COF‐SO_3_^−^]. The new formed broad signal at 401.9 eV originates from [NH_4_]^+^ ions, suggesting the success of ammoniating COF‐SO_3_H by NH_3_·H_2_O. Moreover, due to the formation strong hydrogen bonds between [NH_4_]^+^ and C=O/SO_3_‐ fragment of COF skeleton (Figure 1c), the high resolution O1s spectra for COF‐SO_3_H and [NH_4_]^+^[COF‐SO_3_^−^] shows significant diversity (Figure S4, Supporting Information), where sharp decrease in the signal at 533.2 eV is observed in the ammoniated samples, relative to the pristine COF. The ^13^C CPMAS is also another useful tool to make clear the structure of COF. Figure S5 in the Supporting Information shows the experimental results and the corresponding assignment, fully convincing the β‐ketoenamine‐linked structure in COF‐SO_3_H and [NH_4_]^+^[COF‐SO_3_^−^].^14^

**Figure 2:**
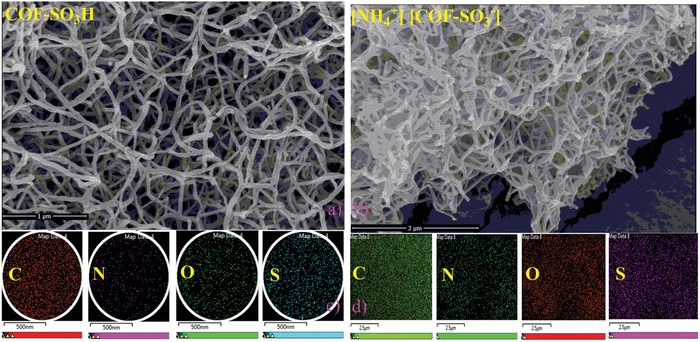
The a,b) SEM images and c,d) EDS mapping results of COF‐SO_3_H and [NH_4_]^+^[COF‐SO_3_^−^], respectively.

The chemostability of [NH_4_]^+^[COF‐SO_3_^−^] is evaluated by PXRD studies (Figure S6, Supporting Information). It is clear that ultrahigh chemostability is observed for [NH_4_]^+^[COF‐SO_3_^−^] under boiling water, strong acid and strong base, as well as γ radiation, suggesting its superior application under rigorous conditions. The permanent porosity of [NH_4_]^+^[COF‐SO_3_^−^] is explored by N_2_ adsorption at 77 K (Figure S7, Supporting Information). The Brunner−Emmet−Teller (BET) surface area and the pore size is estimated to be 110 m^2^ g^−1^ and 1.1 nm, which indicates that relative to COF‐SO_3_H with BET of 306 m^2^ g^−1^ and pore size of 1.2 nm, the loading of NH_4_]^+^ within the COF leads to a decrease in both BET and pore size. The aperture of 1.2 nm for COF‐SO_3_H and 1.1 nm for [NH_4_]^+^[COF‐SO_3_^−^] is comparable with their corresponding estimated values of 1.1 and 1.0 nm from structure data, respectively.

Based on the above results, it is clear that the present porous COF could be a promising adsorbent. Thus, we initially investigated their uranium uptake ability by extracting uranyl from the aqueous solutions with the optimized pH value (Figure S8, Supporting Information). To guarantee adsorption equilibrium, adsorption time is extended to 96 h. The adsorption capacity is obtained in the uranium concentration range of 50–600 ppm, giving 360 mg g^−1^ for COF‐SO_3_H and 851 mg g^−1^ for [NH_4_]^+^[COF‐SO_3_^−^]. The equilibrium adsorption data is well fitted by the Langmuir model with the correlation coefficient higher than 0.99 (**Figure** 3a; Table S1, Supporting Information).[qv: 8e] Accordingly, the calculated adsorption capacity is about 421 mg g^−1^ for COF‐SO_3_H and 869 mg g^−1^ for [NH_4_]^+^[COF‐SO_3_^−^], slightly higher than the experimental value, implying higher theoretical adsorption capacity. The results suggest that ammoniating COF‐SO_3_H by NH_3_·H_2_O can significantly enhance its uranium adsorption capacity. It is worth noting that the adsorption capacity observed in [NH_4_]^+^[COF‐SO_3_^−^] exceeds any reported porous uranium adsorbents (See Table S2 in the Supporting Information),^3^ and is almost 1.6‐fold bigger than the best COF adsorbent,[qv: 11a] 2.2‐fold bigger than the best metal chalcogenide adsorbent,^4^ 2.5‐fold bigger than one of outstanding MOF adsorbents,[qv: 8d] as well as surprisingly 18.1‐fold bigger than the commercial ARSEN‐X^np^ Purolite resin.^15^ Except for the recorded uranium adsorption capacity, [NH_4_]^+^ [COF‐SO_3_^−^] also affords extremely rapid adsorption kinetics as evidenced by the high removal efficiency up to 81% within 1 h contact time for an initial 200 ppm solution. This adsorption also matches well with the pseudo‐second‐order models (the correlation coefficient more than 0.99), indicative of chemical adsorption (Figure 3b; Table S3, Supporting Information). We attribute the ultrahigh adsorption capacity and rapid adsorption kinetics to a synergistic effect from the robust and chemostabile porous framework, ion exchange between [NH_4_]^+^ and U, as well as the strong chemical adsorption site of abundant free‐standing ‐SO_3_^−^ units on the pore wall.

**Figure 3:**
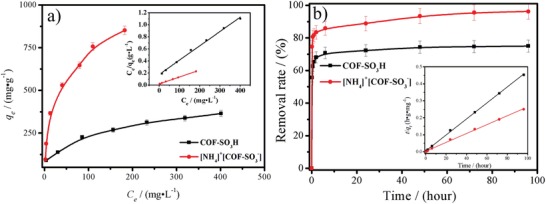
a) Uranium adsorption isotherm and b) kinetics of COF‐SO_3_H and [NH_4_]^+^[COF‐SO_3_^−^]. The insert is the fitting results by means of Langmuir model and the pseudo‐second‐order models, respectively. The fitting parameter is listed in Tables S1 and S3 in the Supporting Information.

To evaluate the affinity of COF toward uranyl, additional adsorption experiments with 50 ppm uranium (V/m = 2000 mL g^−1^) was carried out. After 100 min contact time, almost all uranyl ions were removed. The uranium concentration after 2000 min is 0.01 ppm, suggesting *K*_d_^U^ = 9.8 × 10^6^ mL g^−1^ (**Figure** 4a), which is comparable with some outstanding adsorbents for such use like that of POP‐oNH_2_‐AO with *K*_d_ = 8.36 × 10^6^ mL g^−1^,[qv: 11a] MIPAFs‐11c with *K*_d_ = 6.98 × 10^5^ mL g^−1^,[qv: 11c] and COF‐TpDb‐AO with *K*_d_ = 3.6 × 10^8^ mL g^−1^.[qv: 11b] Generally speaking, a material showing the distribution coefficient *K*_d_^U^ more than 1.0 × 10^4^ mL g^−1^ (a distinguishing standard) is viewed to be a good adsorbent. We attribute the extremely large *K*_d_^U^ value to the unique affinity toward uranyl provided by the free‐standing ‐SO_3_^−^ units on the pore wall of COF. Notably, the residual uranium concentration (10 ppb) is below the acceptable limit of 30 ppb for uranium in potable water defined by the U.S. Environmental Protection Agency (EPA).^16^ To further confirm this claim, selective adsorption of uranyl was carried out from a binary mixed solution containing 50 ppm uranium and other competing metal ions (50 ppm, M = Na^+^, K^+^, Cs^+^, Mg^2+^, Ca^2+^, Sr^2+^, Co^2+^, Ni^2+^, Zn^2+^, Pb^2+^, and Fe^3+^). The selectivity of *S*_U/M_ is calculated from the ratio of *K*_d_^U^:K_d_^M^,^7^ and the results (*S*_U/M_ = 14.8‐821) were described in Figure 4b. Clearly, [NH_4_]^+^[COF‐SO_3_^−^] enables highly selective uranyl adsorption over alkali metals and alkaline earth metals that are major content in seawater, implying promising potential of this material for recovery of uranyl form seawater.

**Figure 4:**
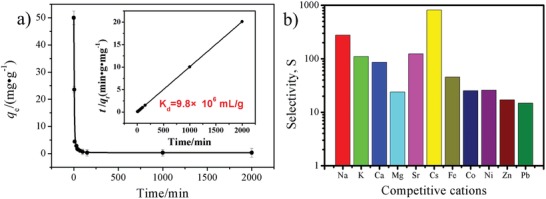
a) The distribution coefficient, *K*_d_^U^ value, calculated from a 50 ppm uranium solution for [NH_4_]^+^[COF‐SO_3_^−^], b) competing uranyl adsorption with the presence of other metal ions and the selectivity calculated from a ratio of *K*_d_^U^:K_d_^M^ for [NH_4_]^+^[COF‐SO_3_^−^]. The insert is the fitting results by means of the pseudo‐second‐order models. The fitting parameter is listed in Table S4 in the Supporting Information.

The robust chemostability, excellent uranium adsorption performance, and the strong uranyl adsorption site from ‐SO_3_^−^ units inspire us to give further investigation under rigorous conditions that is very important for actual industrial application. For example, the nuclear waste solution is highly acid (≈3 m acidity) and generates various radiations such as γ radiation. Seawater often gives pH around 8 and extremely low uranium content about several ppb. To the best of our knowledge, most reported porous adsorbents could not access such goal. As shown in Figure S9 in the Supporting Information, the uranium adsorption under rigorous conditions was executed at pH = 1, 8, and 3 m acidity for a 10 ppm uranium solution. Highly effective uranyl adsorption is observed for both pH = 1 and 8, giving 100% and 98% removal efficiency. Notably, the observation of 48% removal efficiency strongly suggests effective uranyl adsorption even under 3 m acidity (Table S5, Supporting Information). Moreover, we also found almost no decrease in adsorption performance for the material after γ radiation (5 KGy) for eight days (Figure S10, Supporting Information). Furthermore, we tested its usability for the extraction of uranium from seawater, and found that its uranium recovery ability from seawater (total uranium concentration of 10 ppb) is up to 17.8 mg g^−1^, suggesting its superior application in extraction of uranium from seawater.

For practical industrial applications, the recycle use of adsorbent is highly desirable. We first found that the adsorbed uranium in COF samples can be easily desorbed with nearly 100% desorption efficiency by 1 m HCl. Then, the materials after reamination can be reused and this recycle use can be performed for at least six times without any decrease in uranium adsorption performance (Figure S11, Supporting Information). Such excellent recycle use is owing to the high chemostability of COF material and ion‐exchange nature of the present COF, as evidenced by the PXRD results (Figure S12, Supporting Information).

On the other hand, almost all reported uranium adsorbents are based on the evaluation in terms of batch experiments. By contrast, another important experiment such as the breakthrough experiment that is close to meet the practical industrial demand is almost unexplored. In this work, we present the first breakthrough experiment based on [NH_4_]^+^[COF‐SO_3_^−^] adsorbent. Firstly, we carried out the breakthrough experiment with the uranium solution of 10 ppm at pH = 1 flowing over a packed bed of [NH_4_]^+^[COF‐SO_3_^−^] solid with a flow rate of 0.05 mL min^−1^ at room temperature. We found that no uranyl can pass through this packed bed ever after five months. Next, similar breakthrough experiment was implemented with a mixed solution containing 50 ppm uranium and other ions (50 ppm and pH = 5, Na^+^, K^+^, Cs^+^, Mg^2+^, Ca^2+^, Sr^2+^, **Figure** 5; S13, Supporting Information). After about 9 h, Na^+^, K^+^, Cs^+^ starts to pass through this packed bed, and complete breakthrough is observed after 21 h. The next breakthrough ion is Mg^2+^ with complete breakthrough after 70 h, while complete breakthrough of Ca^2+^ and Sr^2+^ is about 173 and 210 h, respectively. Notably, even after one month there is still no observation of uranium that passes through this packed bed. The excellent separation among uranyl, alkali metal, and alkaline earth metal is mainly due to the strong affinity between SO_3_^−^ unit of COF and uranyl ions, but the weak coordination ability of alkali metal, and alkaline earth metal with oxygen‐donor ligand. The exciting results indicate that [NH_4_]^+^[COF‐SO_3_^−^] is a promising material for selective uranyl recovery from nuclear waste solution and seawater.

**Figure 5:**
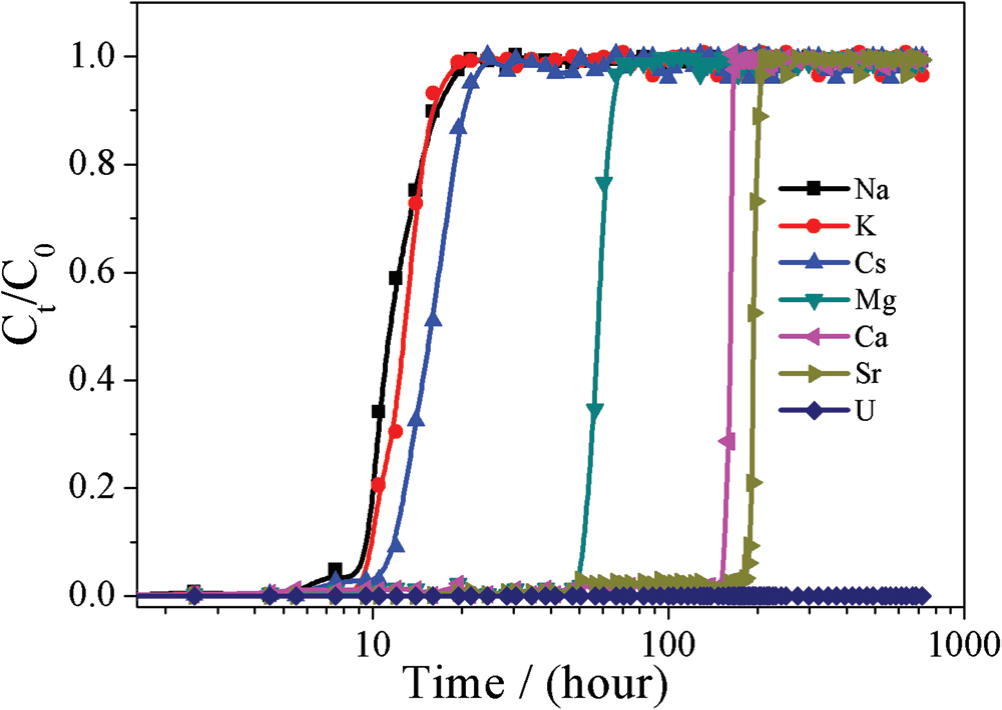
The breakthrough experiments of a mixed solution composed of 50 ppm uranium and 50 ppm other metal ions on the [NH_4_]^+^[COF‐SO_3_^−^] packed bed.

To intuitively reflect the inclusion of uranium on COF adsorbents, both XPS and EDS with elemental distribution mapping were performed. The observation of U_4f_ signals by XPS and a homogeneous uranium distribution by EDS in these reacted samples (COF‐SO_3_H and [NH_4_]^+^[COF‐SO_3_^−^]) directly attested the capture of uranium (Figures S14 and S15, Supporting Information). To check the chemical binding of uranyl in the COF adsorbents, we carried out IR studies, and new peaks at 926 and 918 cm^−1^ was observed for the COF‐SO_3_H and [NH_4_]^+^[COF‐SO_3_^−^] samples (Figure S16, Supporting Information), respectively, which are assigned to the antisymmetric vibration of uranyl ions.^11^ Thus, relative to the corresponding peak at 960 cm^−1^ in UO_2_(NO_3_)_2_·6H_2_O, the peaks observed in the present COF adsorbents show big redshift, indicative of strong coordination interactions between uranyl ion and ‐SO_3_^−^ groups of COF. Moreover, as evidenced in the IR spectra for [NH_4_]^+^[COF‐SO_3_^−^] adsorbent with the disappearance of peaks at 3155 cm^−1^ for the uranyl‐loaded samples, ion exchange between [NH_4_]^+^ and uranyl ion can be deduced, which can be further confirmed in the XPS signals for N element (Figure S17, Supporting Information).

To gain further insight into the uranyl adsorption mechanism on [NH_4_]^+^[COF‐SO_3_^−^], the density functional theory (DFT) calculation^17^ was carried out for the uranyl‐loaded samples and the DFT optimized structure is shown in **Figure** 6. The experimental PXRD patterns match well with the data from simulated structure (Figure S18, Supporting Information). The coordination surrounding in the uranyl‐loaded structure for [NH_4_]^+^[COF‐SO_3_^−^] samples is composed of two ‐SO_3_^−^ oxygen atoms, two NO_3_^−^ oxygen atoms, and two uranyl oxygen atoms, creating the typical octahedral geometry. The U‐O(SO_3_^−^) bond lengths are comparable with the reported U‐O(SO_3_^−^) values^13^ in the literature. The ‐SO_3_^−^ unit within the COF affords the chelate coordination mode with uranyl ion (similar to the reported mode in the literature^13^), thus providing high affinity toward uranyl ion, well consistent with the experimental results. The adsorbed NO_3_^−^ ions not only balance the charge but also bind to uranyl ion in a chelate mode. The existence of NO_3_^−^ ions in the uranyl‐loaded samples can be supported by ^15^N CPMAS solid‐state NMR spectroscopy (Figure S19, Supporting Information). In this regard, the remarkable uranyl adsorption performance observed in [NH_4_]^+^[COF‐SO_3_^−^] is due to first ion exchange between [NH_4_]^+^ and uranyl ions and next strong coordination interactions between ‐SO_3_^−^ oxygen atoms and uranyl ions.

**Figure 6:**
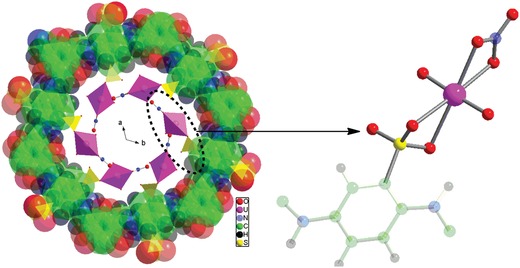
View of the DFT optimized uranyl‐loaded structure for the samples of [NH_4_]^+^[COF‐SO_3_^−^]. The highlight is the coordination surrounding around uranyl ions.

To further explain why ammoniating COF can largely enhance uranyl adsorption performance, a comparison in the aspect of the free energy was implemented. As shown in Figure 1, the NH_4_^+^ cations replaced the position of H^+^, and were adsorbed near sulfonic functional groups by triple hydrogen bonds with two sulfonic oxygen atoms (H_H‐O_ = 1.81 Å) and one carbonyl oxygen atom (H_H‐O_ = 1.77 Å). Thus, it results in relatively lower free energy of −0.76 eV for ammoniating COF, and further indicates that the structure of [NH_4_]^+^[COF‐SO_3_^−^] is stable than the pristine COF. As discussed above, the uranyl adsorption on [NH_4_]^+^[COF‐SO_3_^−^] is in this way that first the NH_4_^+^ cations were rapidly displaced by (UO_2_)NO_3_^+^ cations, leading to the release of NH_4_NO_3_, and then the (UO_2_)NO_3_^+^ cations were binded by sulfonic unit. Accordingly, the thermodynamic free energy for adsorbing (UO_2_)NO_3_^+^ cation is −0.39 eV, which also means that the adsorption process was spontaneous and the uranyl‐loaded structure was more stable. By contrast, higher free energy of −0.27 eV was generated for the direct adsorption of (UO_2_)NO_3_^+^ cation by using COF‐SO_3_H materials was −0.27 eV, consequently leading to relatively poor uranyl adsorption ability for COF‐SO_3_H, in contrast to [NH_4_]^+^[COF‐SO_3_^−^] (Figure S20, Supporting Information).

In summary, we have demonstrated in this work a new concept for designing uranium adsorbent through a combined approach composed of both ion exchange and coordination interaction. Thereout, the resultant porous adsorbent affords not only ultrahigh chemostability in strong acid, alkali, and boiling water, as well as γ radiation, but also record‐breaking uptake capacity up to 851 mg g^−1^. Moreover, several other remarkable advantages are also observed, including in the facile syntheses of adsorbent, rapid kinetics, broad pH active range even under rigorous conditions, high affinity with *K*_d_^U^ = 9.8 × 10^6^ mL g^−1^, excellent selectivity, and good recycle use. All these results suggest its superior application in the uranium removal and recovery. This new concept was further explained by DFT calculation. And it is shown that in this system ammoniating material can reduce free energy and thus enhance adsorption capacity. This proof‐of‐concept study outlines a new direction for designing high‐performance uranium adsorbent.

## Conflict of Interest

The authors declare no conflict of interest.

## Supporting information

Supplementary
