# Correction to “Ammoniating Covalent Organic Framework (COF) for High‐Performance and Selective Extraction of Toxic and Radioactive Uranium Ions”

**DOI:** 10.1002/advs.202505740

**Published:** 2025-05-28

https://doi.org/10.1002/advs.201900547

Regrettably, an error occurred during the manuscript revision process; we mistakenly uploaded the incorrect Supporting Information file with our previous submission. The previously published version of the Supporting Information file featured some wrong/incomplete information as follows. All the conclusions in the original manuscript remain unchanged. The authors regret this scenario and apologize for any inconvenience caused.

**Detailed list of corrections**:

**Main text** (page 7, left)

Figure S13 (Supporting Information) has not been cited in the manuscript file, and it was mislabeled as Figure S7 (Supporting Information), we have added it into the main text as follows:

“On the other hand, almost all reported uranium adsorbents are based on the evaluation in terms of batch experiments. By contrast, another important experiment, such as the breakthrough experiment, that is close to meet the practical industrial demand is almost unexplored. In this work, we present the first breakthrough experiment based on [NH_4_]^+^[COF‐SO_3_^−^] adsorbent (Figure S13, Supporting Information). First, we carried out the breakthrough experiment with the uranium solution of 10 ppm at pH 1 flowing over a packed bed of [NH_4_]^+^[COF‐SO_3_^−^] solid with a flow rate of 0.05 mL min^−1^ at room temperature.” And the numbering of Figure S7 (Supporting Information) has been corrected to Figure S13 (Supporting Information).

## Supporting Information

Figure S3 needs a correction due to an error in the Supplementary Information: the correct Figure S3 was missing, and Figure S4 was mistakenly labeled as Figure S3.


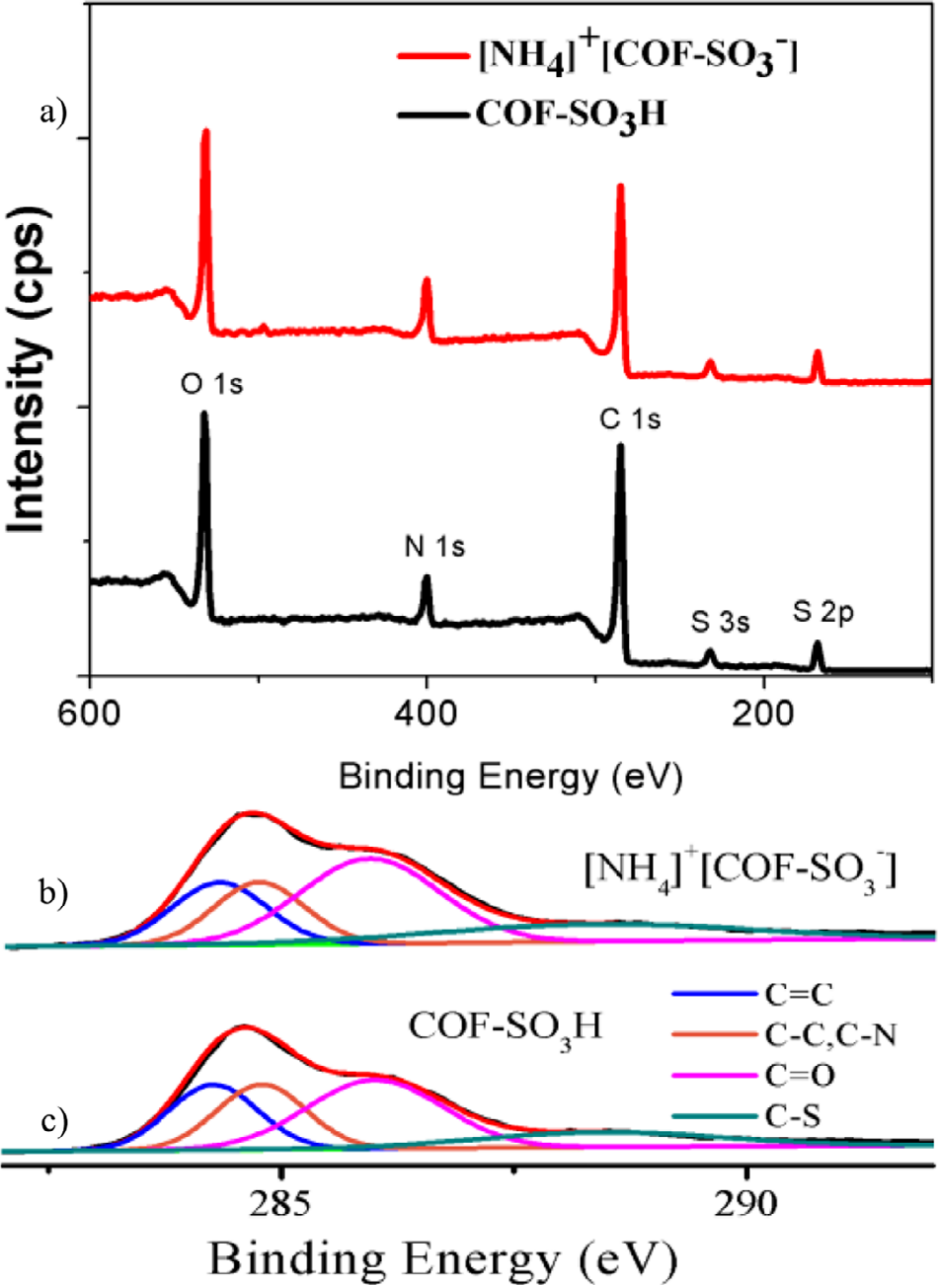


**Figure S3**. The XPS results of COF‐SO_3_H and [NH_4_]^+^[COF‐SO_3_^−^]: a) the complete survey XPS plots; b) and c) the high resolution C1s spectra.

The numbering of Figure S3 has been corrected to Figure S4.


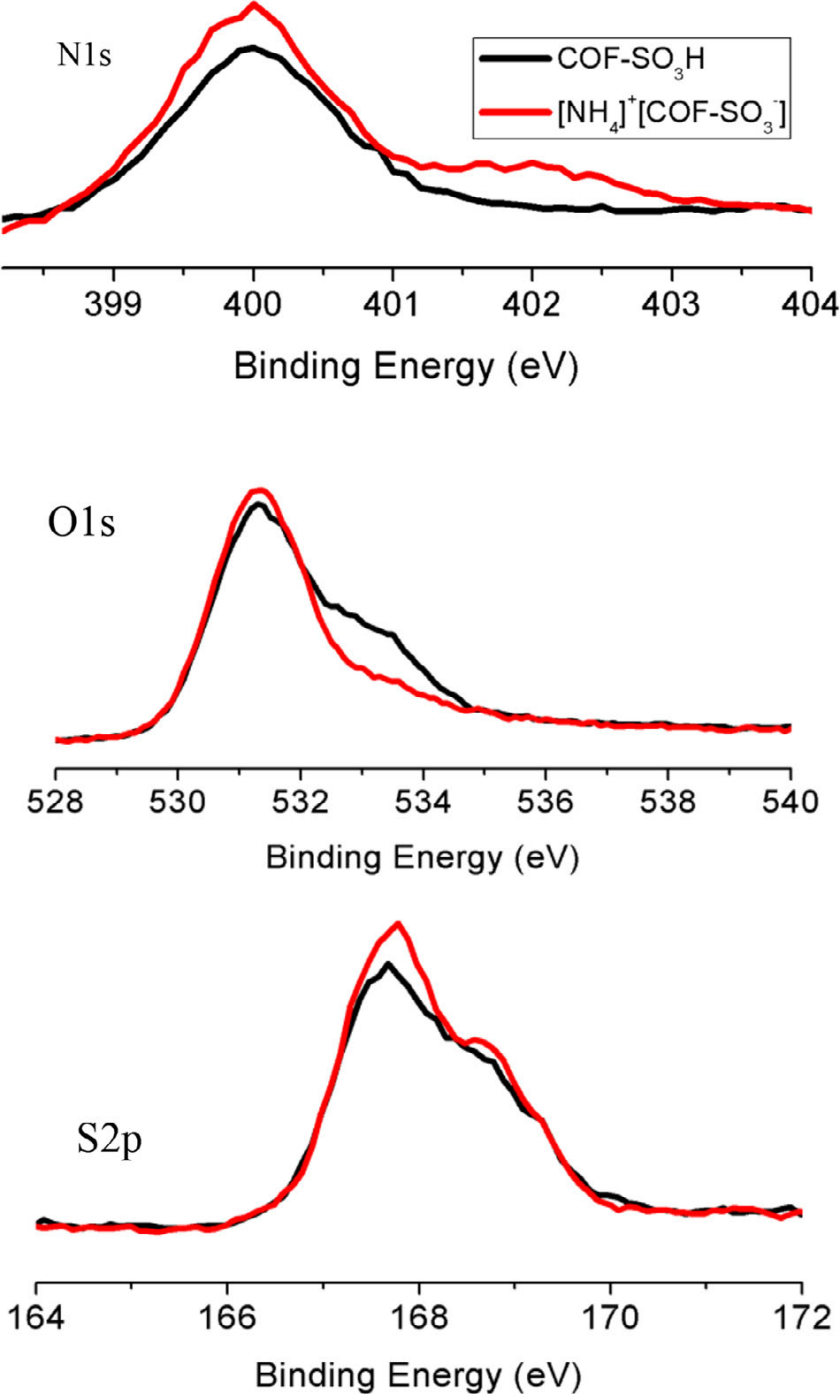


**Figure S4**. The high‐resolution spectra of N1s, O1s, and S2p of COF‐SO_3_H and [NH_4_]^+^[COF‐SO_3_^−^].

Figure S4 needs a correction. The numbering of Figure S4 has been corrected to Figure S5. Additionally, in the Supporting Information, the fragment structure of the COF‐SO_3_H was drawn incorrectly, and the peaks in the ^13^C CP‐MAS spectrum of COF‐SO_3_H and [NH_4_]^+^[COF‐SO_3_^−^] were also mislabeled, it has been corrected as follow:


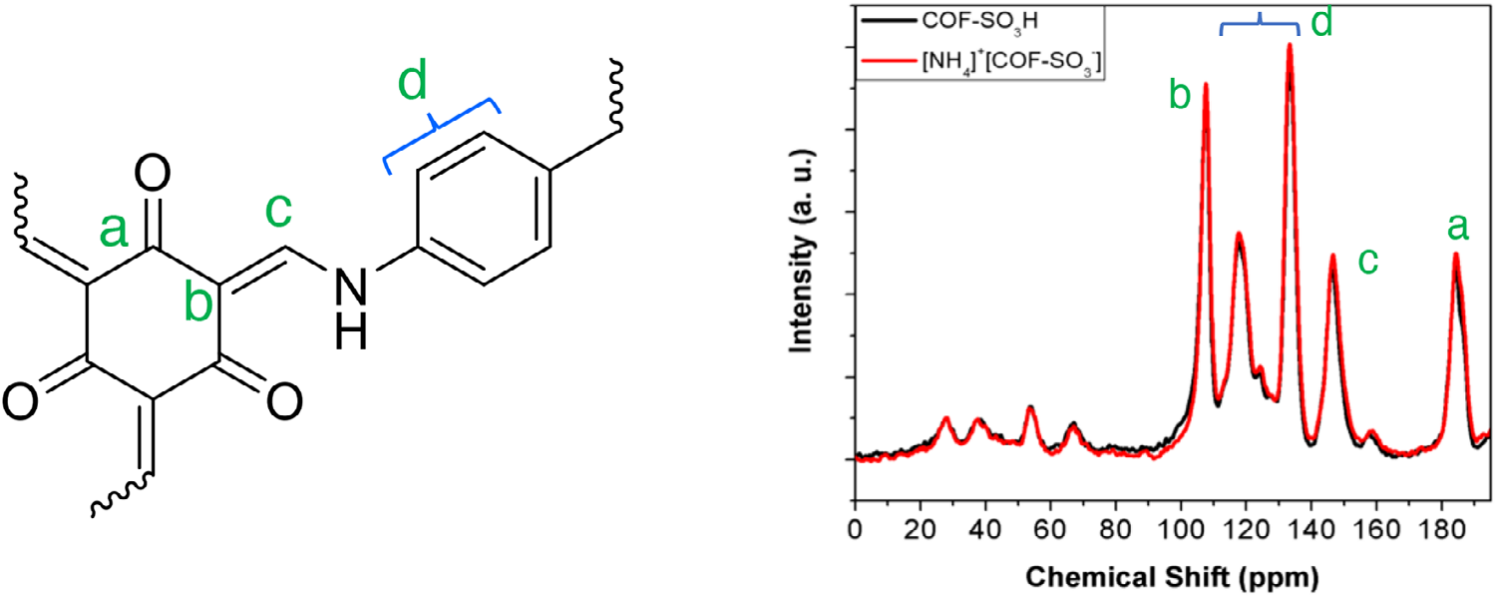


**Figure S5**. ^13^C CP‐MAS spectrum of COF‐SO_3_H and [NH_4_]^+^[COF‐SO_3_^−^].

The correct Figure S6 was missing in the Supporting Information, it has been added as follow:


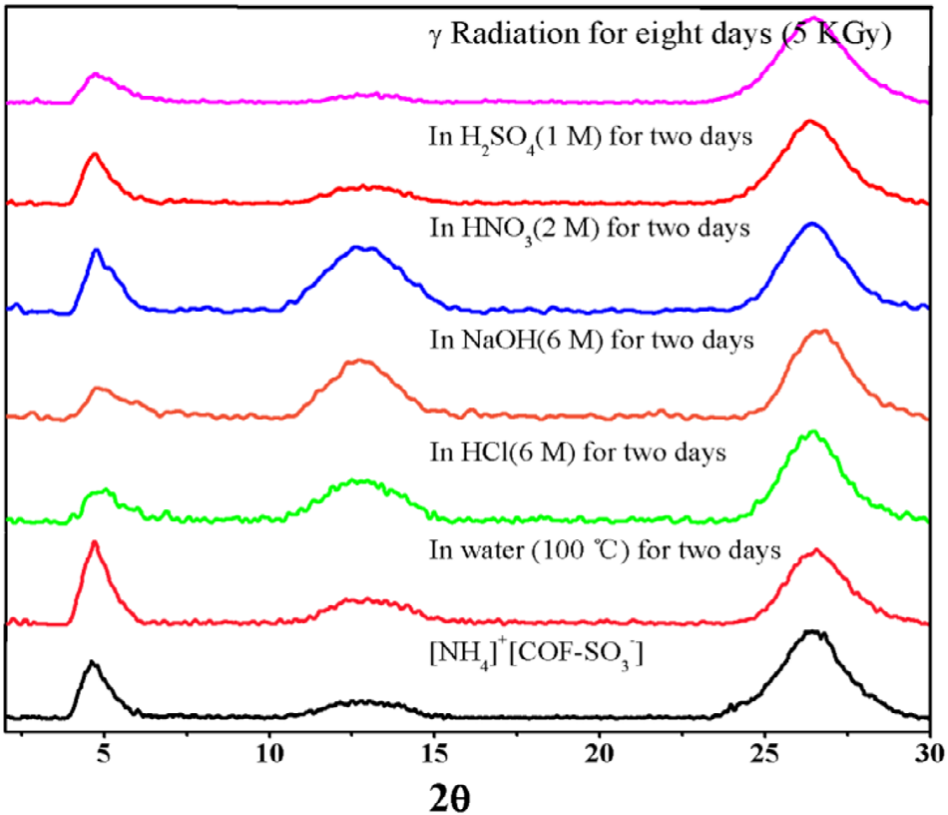


**Figure S6**. The chemostability of [NH_4_]^+^[COF‐SO_3_^−^] under boiling water, strong acid and strong base, as well as γ radiation tracked by PXRD patterns.

The correct Figure S7 was missing in the Supporting Information, it has been added as follow:


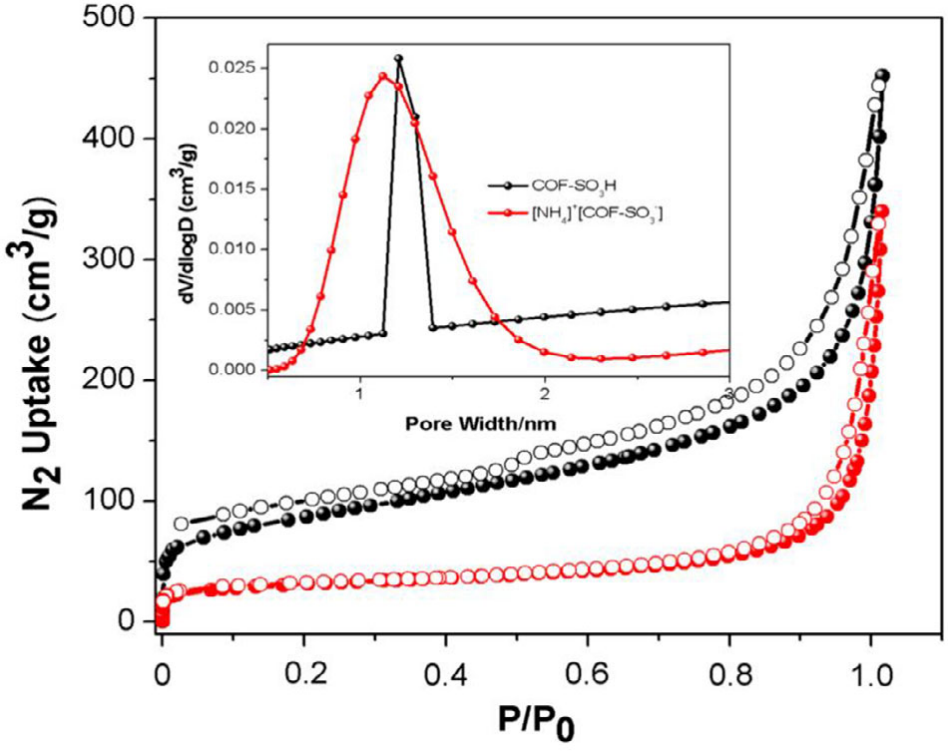


**Figure S7**. The N_2_ adsorption at 77 K for COF‐SO_3_H and [NH_4_]^+^[COF‐SO_3_^−^] with the insert of distribution of pore size.

The numbering of Figure S5 has been corrected to Figure S8.


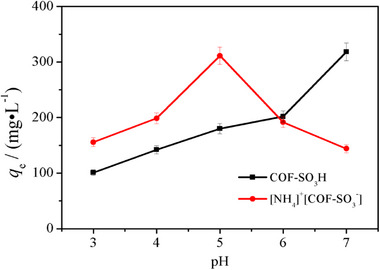


**Figure S8**. The pH‐dependent U adsorption on COF‐SO_3_H and [NH_4_]^+^[COF‐SO_3_^−^] materials.

The correct Figure S9 was missing in the Supporting Information, it has been added as follow:


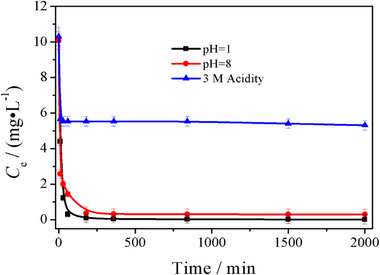


**Figure S9**. U adsorption of [NH_4_]^+^[COF‐SO_3_^−^] under pH = 1, 8 and 3 M acidity.

Figure S10 was missed in the Supporting Information, it has been added as follow:


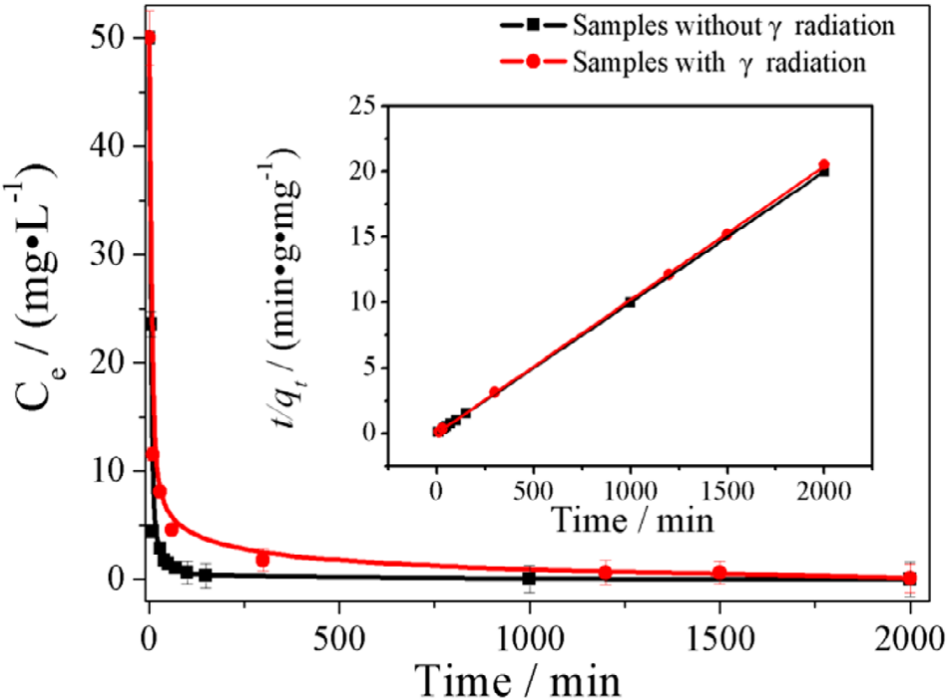


**Figure S10**. A comparison in the U adsorption for the samples of [NH_4_]^+^[COF‐SO_3_^−^] without or with γ radiation. The insert is the fitting results by means of the pseudo‐second‐order models. The fitting parameter is listed in Table S4 and S5.

The correct Figure S11 was missed in the Supporting Information, it has been added as follow:


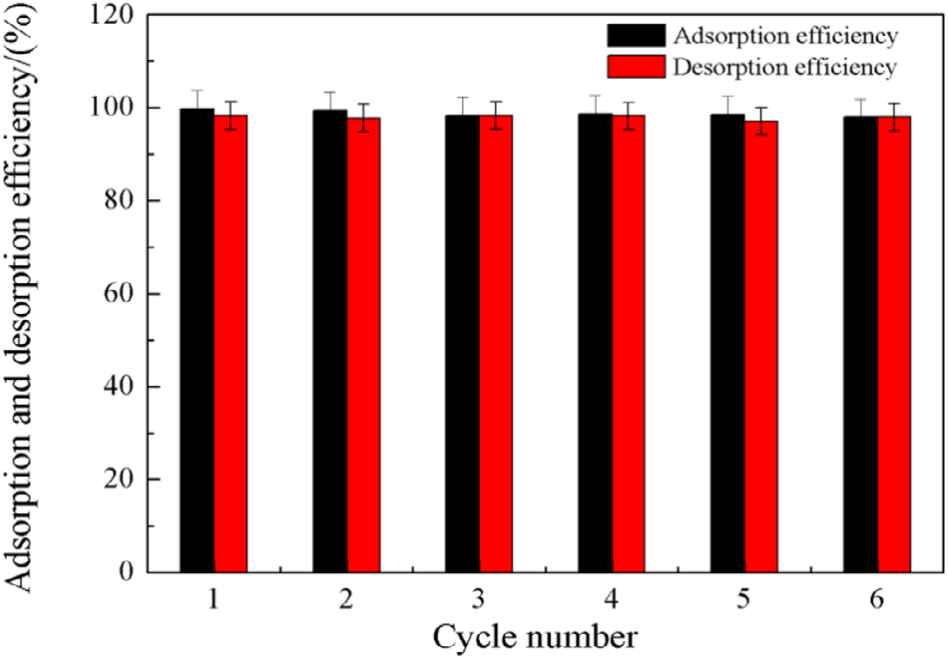


**Figure S11**. The recycle use of [NH_4_]^+^[COF‐SO_3_^−^] for U adsorption from a 50 ppm U solution.

The correct Figure S12 was missed in the Supporting Information, it has been added as follow:


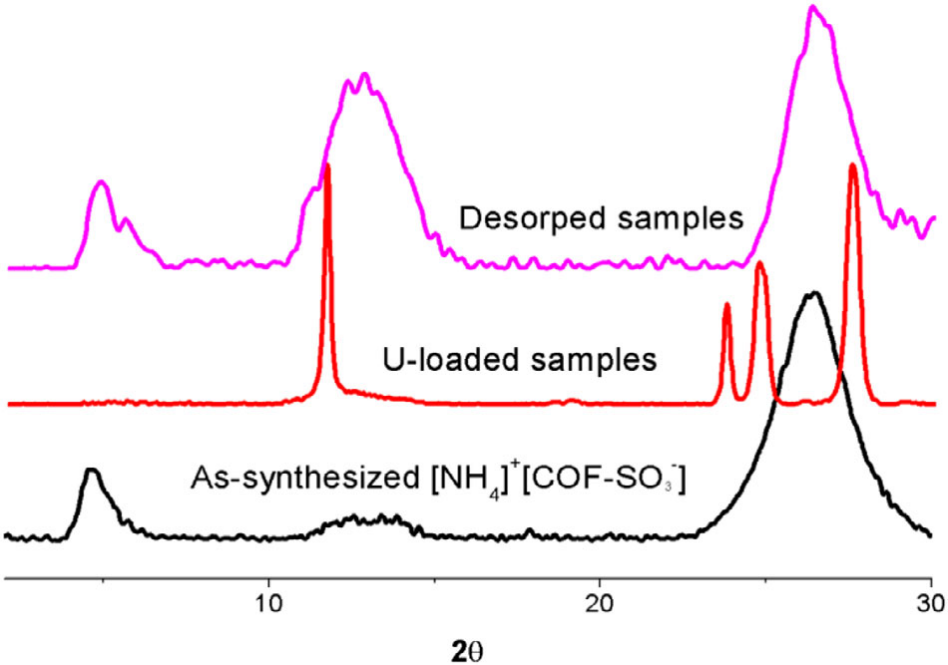


**Figure S12**. A comparison of PXRD patterns among these samples included in as‐synthesized [NH_4_]^+^[COF‐SO_3_^−^] samples, the samples after U adsorption, and the samples after desorption of U by HCl.

The numbering of Figure S7 has been corrected to Figure S13.


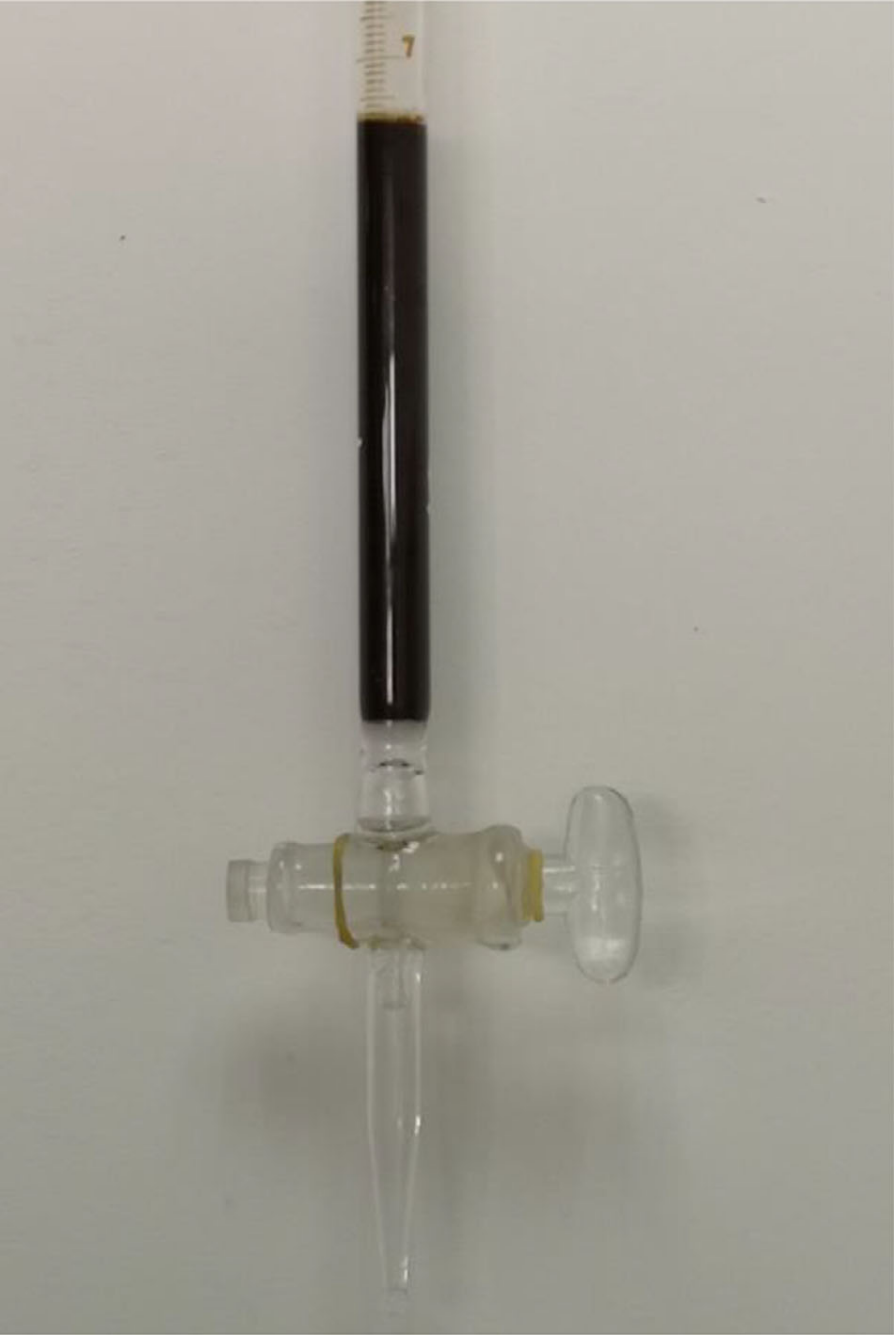


**Figure S13**. The packed bed by means of [NH_4_]^+^[COF‐SO_3_^−^] samples used in this work.

The numbering of Figure S8 has been corrected to Figure S14.


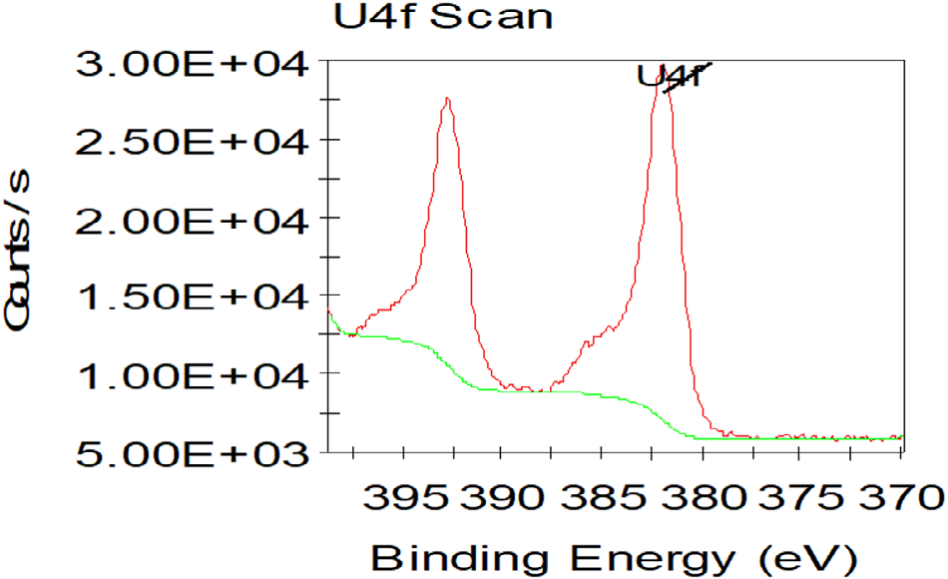


**Figure S14**. The high resolution spectra of U_4f_ for the samples of [NH_4_]^+^[COF‐SO_3_^−^] after loading of U.

The numbering of Figure S9 has been corrected to Figure S15.


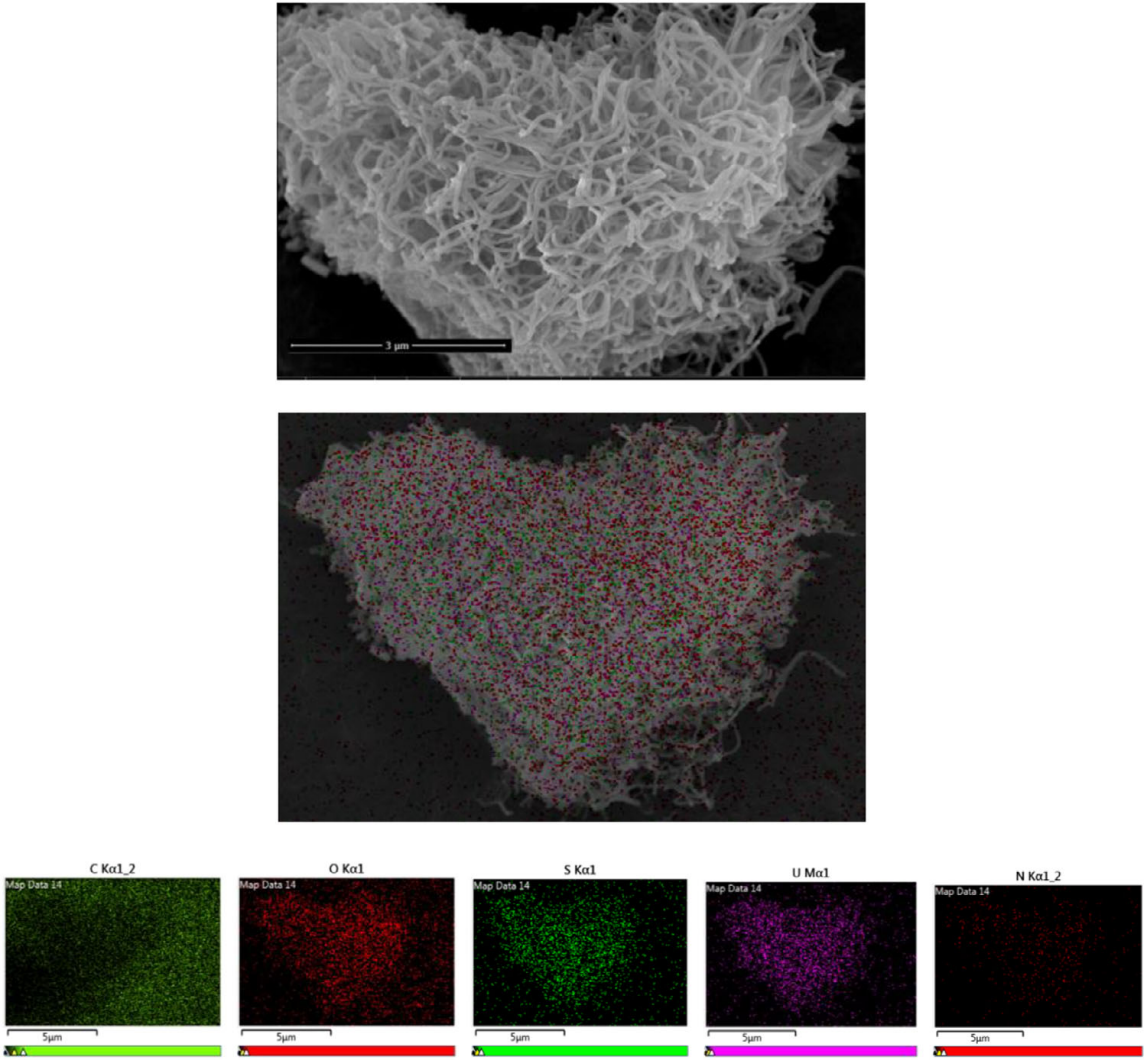


**Figure S15**. SEM and EDS mapping image of the samples of [NH_4_]^+^[COF‐SO_3_^−^] after loading of U.

The numbering of Figure S10 has been corrected to Figure S16.


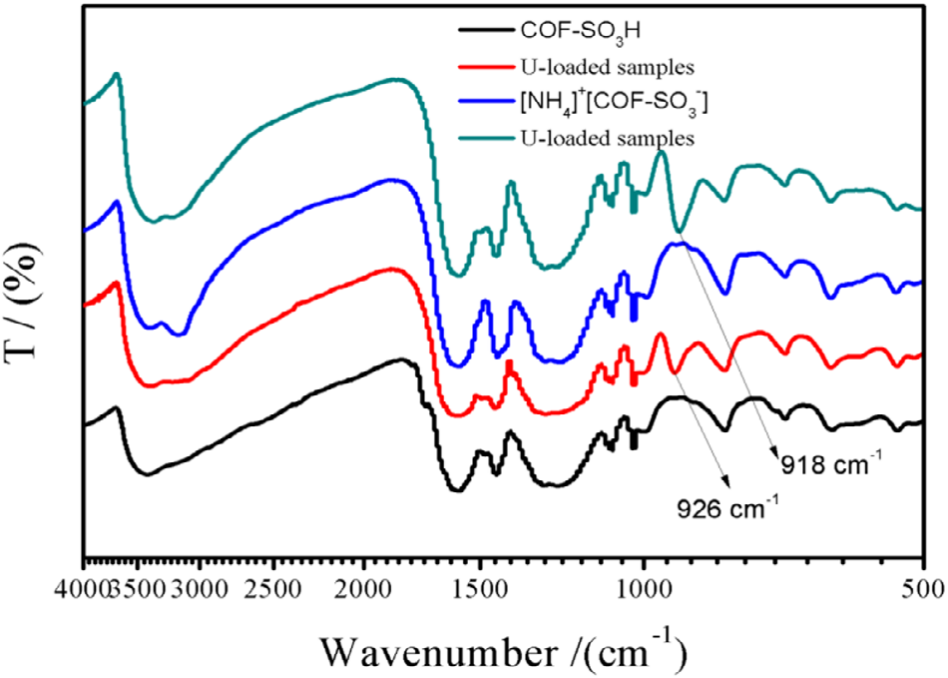


**Figure S16**. The IR bonds of COF‐SO_3_H and [NH_4_]^+^[COF‐SO_3_^−^] samples and their corresponding U‐loaded samples.

The numbering of Figure S11 has been corrected to Figure S17.


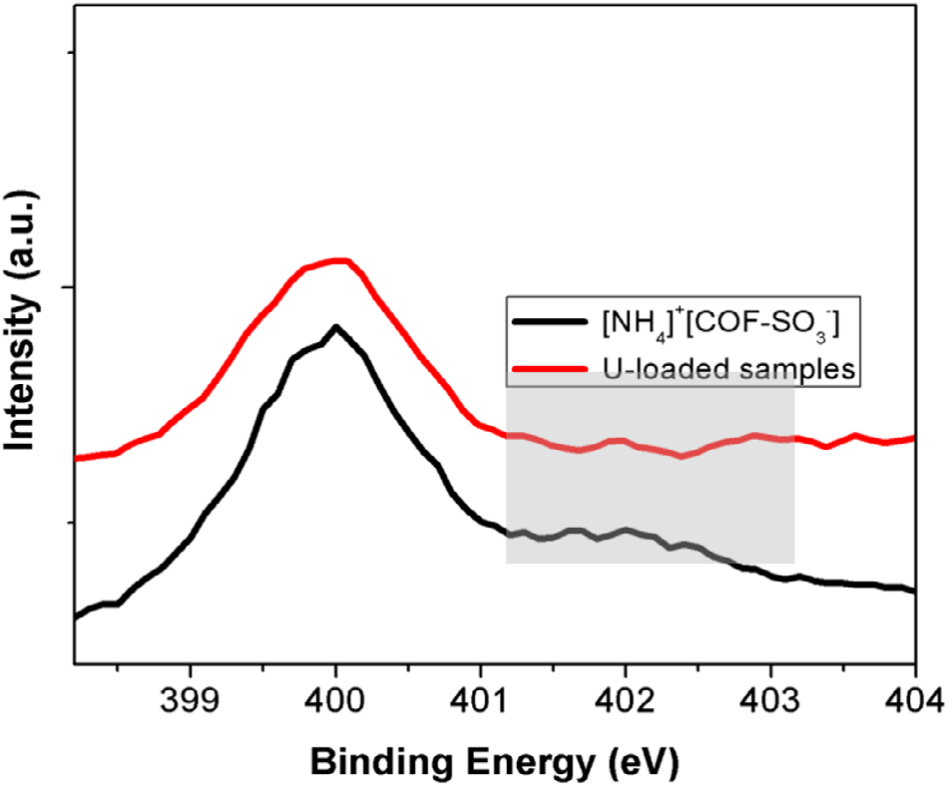


**Figure S17**. A comparison in the high resolution spectra of N1s for [NH_4_]^+^[COF‐SO_3_^−^] samples and samples after loading of U.

The numbering of Figure S12 has been corrected to Figure S18.


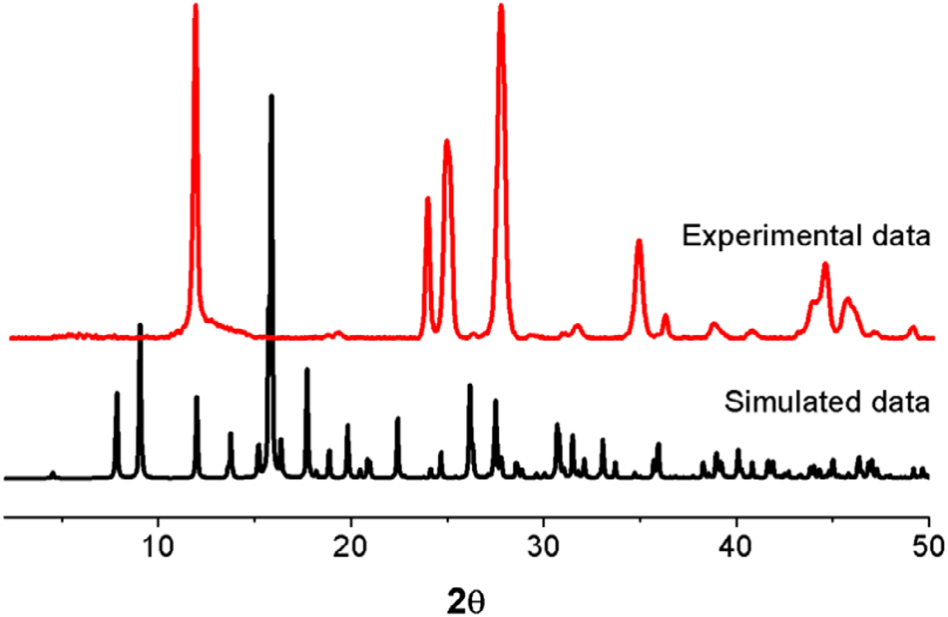


**Figure S18**. The experimental and simulated PXRD patterns for the samples of [NH_4_]^+^[COF‐SO_3_^−^] after loading of U.

The numbering of Figure S13 has been corrected to Figure S19.


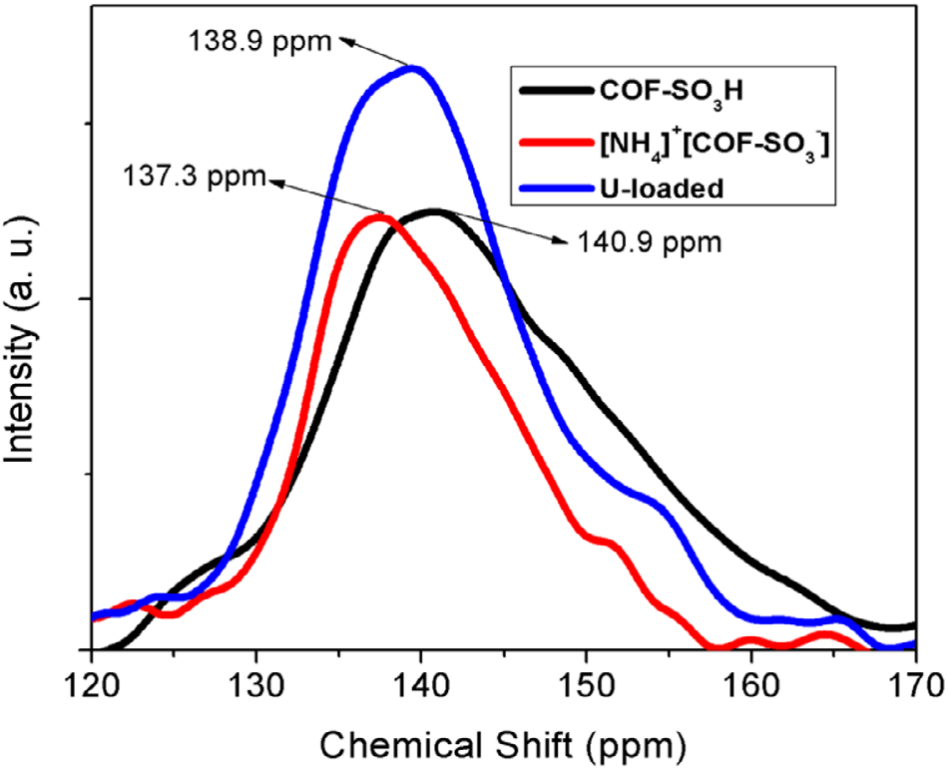


**Figure S19**. A comparison in the ^15^N CP‐MAS spectrum of COF‐SO_3_H, [NH_4_]^+^[COF‐SO_3_^−^], and the samples of [NH_4_]^+^[COF‐SO_3_^−^] after loading of U.

The numbering of Figure S13 has been corrected to Figure S20.


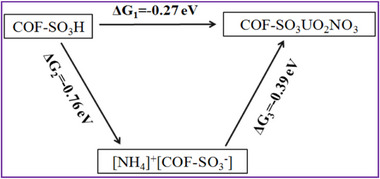


**Figure S20**. The free energy for both the pristine COF (COF‐SO_3_H) and ammoniated COF ([NH_4_]^+^[COF‐SO_3_^−^]) for adsorbing (UO_2_) NO_3_^+^ cation.

