# Supplementary material for: Ammoniating Covalent Organic Framework (COF) for High‐Performance and Selective Extraction of Toxic and Radioactive Uranium Ions
Source: Adv Sci (Weinh). 2019 Jun 27;6(16):1900547. doi: 10.1002/advs.201900547 (PMC6702651; doi:10.1002/advs.201900547)
Supplement: Supplementary file 1 — Supplementary [file ADVS-6-1900547-s001.pdf]

Copyright WILEY-VCH Verlag GmbH & Co. KGaA, 69469 Weinheim, Germany, 2019.

ADVANCED SCIENCE
Open Access

# Supporting Information

for *Adv. Sci.,* DOI: 10.1002/advs.201900547

# Ammoniating Covalent Organic Framework (COF) for High-Performance and Selective Extraction of Toxic and Radioactive Uranium Ions

*Xiao Hong Xiong, Zhi Wu Yu, Le Le Gong, Yuan Tao, Zhi Gao, Li Wang, Wen Hui Yin, Li Xiao Yang, and Feng Luo**

Copyright WILEY-VCH Verlag GmbH & Co. KGaA, 69469 Weinheim, Germany, 2019.

Supporting Information

# Ammoniating Covalent Organic Framework for High-Performance and Selective Extraction of Toxic and Radioactive Uranium Ions

*Xiao Hong Xiong,*[a†] *Zhi Wu Yu,*[b†] *Le Le Gong,*[a†] *Yuan Tao,*[a] *Zhi Gao,*[a] *Li Wang,*[a] *Wen Hui Yin,*[a] *Li Xiao Yang,*[a] *and Feng Luo*[a]*

[a]State key Laboratory of Nuclear Resources and Environment, School of Biology, Chemistry and Material Science, East China University of Technology, Fuzhou, Jiangxi 344000, China

[b]High Magnetic Field Laboratory, Chinese Academy of Sciences, Hefei 230031, Anhui, China

E-mail: ecitluofeng@163.com

**Experimental Section**

**Synthesis of 2,4,6-Triformylphloroglucinol.** Hexamethylenetetraamine (15.098 g, 108 mmol) and phloroglucinol (6.014 g, 49 mmol) was added into 90 mL trifluoroacetic acid under $N_2$. The solution was heated at 100℃ for 2.5 h. Then 150 mL of 3 M HCl was added and the solution was heated at 100℃ for 1 h. After cooling to room temperature, the solution was filtered through Celite, extracted with 350 mL dichloromethane, dried over magnesium sulfate, and filtered. Rotary evaporation of the solution afforded 1.23g (5.87mmol ,11%) of an off-white powder. The pure sample was obtained by sublimation. $^1$H NMR (400 MHz, $CDCl_3$) data indicated near 99% purity, giving 14.12 (s, 3H, OH), 10.15 (s, 3H, CHO) ppm. Element analysis: calculated value C,51.44; H, 2.88; N, 0.00; Found: C 51.38; H 2.80; N, 0.00.

**Synthesis of 2,5-diaminobenzenesulfonic acid**. p-Phenylenediamine (2.7g, 25 mmol) was added into 20% fuming sulfuric acid (33g, 66 mmol). The solution was heated at 145℃ for 7 h. Then 4 mL deionized water was added, and the solution was heated at 135℃ for 3 h. Cooling to 30℃ for static crystallization for 2 h. 2 mL deionized water was added and the pH value is adjusted to 4-5 with 30% sodium hydroxide. Then cooled to 10℃ and filtered to obtain a product 3.8g with purity greater than 98%. $^1$H NMR (400 MHz, DMSO) data for 2,5-diaminobenzenesulfonic acid: 7.39 (s,

1H), 6.94 (d, $J$ = 8.5 Hz, 1H), 6.79 (d, $J$ = 8.5 Hz, 1H). Element analysis: calculated value C, 38.29; H, 4.28; N, 14.88; S, 17.04; Found: C 38.22; H 4.32; N, 14.83; S, 17.00.

**Synthesis of COF-$SO_3$H.** 0.3 mmol (63mg) of 2,4,6-Triformylphloroglucinol, 0.45 mmol (84.7mg) 2,4,6-Triformylphloroglucinol was added into a Pyrex tube with 1.5 mL butyl alcohol and 1.5mL 1,2-dichlorobenzene. The mixture was sonicated for 20 min, followed by addition of 0.5 mL of 3 M aqueous acetic acid. After that, the tube was degassed by freeze-pump-thaw cycles for three times, sealed under vacuum and heated at 120 °C for 3 days. The reaction mixture was cooled to room temperature and washed with deionized water, dimethylacetamide and acetone. The resulting dark red powder was dried at 120 °C under vacuum for 12 hours.

**Synthesis of $[NH_4]^+$[COF-$SO_3^-$].** 50 mg COF-$SO_3$H was added into 20mL glass bottles with 10 mL (1%) ammonium hydroxide and stir for 30 hours. Then the resultant materials were washed by deionized water, acetone and methanol. The resulting dark red powder was dried at 60 °C under vacuum for 12 hours.

**The activation of samples for $N_2$ test.** 100 mg COF-$SO_3$H or $[NH_4]^+$[COF-$SO_3^-$] samples were immerge in $CH_3OH$ (30 mL) for three days with changing $CH_3OH$ three times every day. Then the samples were transferred to Belsorp-max and 110°C was used to activate the samples about 24 h.

**Materials and general methods.** Reagents and solvents were commercially available (Alfa) and were used without further purification. X-ray powder diffraction were collected by a Bruker AXSD8 Discover powder diffractometer at 40 kV, 40 mA for Cu Kλ ( λ= 1.5406 Å). The simulated powder patterns were calculated by Mercury 1.4. Infrared Spectra (IR) were measured by a Bruker VERTEX70 spectrometer in the 500-4000 $cm^{-1}$ region. The gas adsorption isotherms were collected on a Belsorp-max. Ultrahigh-purity-grade (>99.999%) $N_2$ gases were used during the adsorption measurement. SEM and EDS measurements were carried out using a Hitachi S-4800 microscope. The analyses of concentrations of metal ions in the solution was carried out by ThermoFisher iCapQ ICP-MS and ThermoFisher iCap7600 ICP-OES instruments. Elemental analyses of C, H, N, and S were carried out on a German Elementary Vario EL III instrument. X-ray photoelectron spectra (XPS) were collected by Thermo Scientific ESCALAB 250 Xi spectrometer. The NMR in solution was carried out on Bruker VANCEIIIHD500. The NMR in solide was carried out on a Bruker 400MHz WB solid-state NMR spectrometer.

**Uranium uptake *via* batch experiments.** U(VI) solution was prepared by dissolving uranyl nitrate ($UO_2(NO_3)_2\cdot 6H_2O$, analytical reagent) in deionized water. The pH value is adjusted by $HNO_3$ (1 M) and NaOH (1 M). Adsorption temperature is 298 K.

In pH-dependent experiments, the U solution with pH=3-7 was adjusted by HCl (1 M) and NaOH (1 M). The dose of adsorbent is 10 mg, while the U solution is 20 mL and the contact time is 96 h.

In isotherm experiments, the U solution with initial concentration of 50-600 ppm and pH=5 was used. The dose of adsorbent is 10 mg, while the U solution is 20 mL and the contact time is 96 h.

In kinetics experiments, the U solution with initial concentration of 200 ppm and pH=5 was used. The dose of adsorbent is 10 mg, while the U solution is 20 mL.

In determining affinity experiments, the U solution with initial concentration of 50 ppm and pH=5 was used. The dose of adsorbent is 10 mg, while the U solution is 20 mL.

In selective adsorption experiments, a binary mixed solution contains both U and other metal ions respectively with initial concentration of 50 ppm and pH=5 was used. The dose of adsorbent is 10 mg, while the solution is 20 mL.

In the U uptake under rigorous conditions, the U solution with initial concentration of 10 ppm and pH=1, 8, and 3 M HCl was used. The dose of adsorbent is 10 mg, while the solution is 20 mL.

For the samples after γ radiation (5 Gy) for eight days, the U adsorption experiments was carried out in the U solution with initial concentration of 50 ppm and pH=5. The dose of adsorbent is 10 mg, while the solution is 20 mL.

For extraction of U from seawater, first the U concentration of seawater is concentrated to 10 ppb by vaporizing part of water from seawater. Then, 10 mg $[NH_4]^+[COF\text{-}SO_3^-]$ adsorbent was added into a 30 L concentrated seawater with U concentration of 10 ppb for 7 days contact time.

**Uranium uptake *via* breakthrough experiments.** As shown in Figure S17, the packed bed was prepared by filling $[NH_4]^+[COF\text{-}SO_3^-]$ adsorbent (2 g) into a glass tube with inner aperture of 0.8 mm and length of 1 m. The experiment was carried out at room temperature and a flow rate of 0.05 ml/min for the outflow solution was used. Two distinct solutions were used to evaluate the adsorption performance of $[NH_4]^+[COF\text{-}SO_3^-]$. One is a U solution with initial concentration of 10 ppm and pH=5. The other is a mixed solution (pH=5) composed of U, $Na^+$, $K^+$, $Cs^+$, $Mg^{2+}$, $Ca^{2+}$, and $Sr^{2+}$and their initial concentration is 50 ppm.

**Some related calculation and fitting in this U adsorption experiments.** The adsorption amount, $Q_e$ (mg/g), was calculated by the difference of the U(VI) equilibrium concentration before and after adsorption (see equation 1). All experimental data was the average of triplicate determinations and the relative errors were controlled within ±5%.

$$Q_e = \frac{(C_0 - C_e) \times V}{m} \qquad (1)$$

In equation (1), $c_0$ (mg/L) and $c_e$ (mg/L) are the initial concentration and equilibrium concentration of uranium in the solutions, respectively; $V$ (mL) is the volume of testing solution and $m$ (mg) is the amount of sorbent.

The adsorption kinetics was analyzed by simplified kinetic models such as the pseudo-first-order and pseudo-second-order, through the following two equations,

$$\ln(Q_e - Q_t) = \ln Q_e - k_1 \qquad (2)$$

$$\frac{t}{Q_t} = \frac{1}{k_2 \times q_e{}^2} + \frac{t}{Q_e} \qquad (3)$$

Where $Q$e (mg/g) and $Q$t (mg/g) are the quantity of the adsorbed U(VI) at equilibrium and at $t$ time, respectively, and $K_1$ ($min^{-1}$)/$K_2$[g/(mg•min)] is the pseudo-first/second-order sorption rate constant that is deduced from the slope of the plot of $t/Q$t versus $t$.

The isotherm data was fitted by Langmuir and Freundlich models *via* the following two equations,

$$\frac{c_e}{Q_e} = \frac{c_e}{Q_m} + \frac{1}{k_L Q_m} \qquad (4)$$

$$\ln Q_e = \ln k_F + \frac{1}{n}\ln c_e \qquad (5)$$

Where $Q_e$ (mg/g) is the amount adsorbed at equilibrium and $C_e$ (mg/L) is the equilibrium concentration; $Q_m$(mg/g) is the maximum adsorption amount; $K_L$ (L/mg) is an equilibrium constant related to the binding strength; $n$ and $K_F$ (L/mg) are Freundlich constants which are indicators of the adsorption capacity and adsorption intensity, respectively.

The $K_d$ value and selectivity ($S$) is calculated from the following two equations,

$$K_d = \frac{V}{m}\frac{(C_0 - C_e)}{C_e} \times 10^3 \qquad (6)$$

$$S = \frac{K_d^U}{K_d^M} \qquad (7)$$

where the unit for $K_d$ value is mL/g.

**DFT calculation.** A series of models were established for optimizing the initial structure of COF with $-SO_3H$ function groups in frameworks, as well as for simulating the adsorption behavior of $NH_4^+$ and $(UO_2)NO_3^+$cations in COF pores. The periodic density functional theory calculations were performed by using the Vienna Ab initio Simulation Package (VASP) code. The Perdew-Burke-Ernzerhof (GGA-PBE) functional was utilized to calculate the exchange-correlation energy, and the project-augmented wave generalized gradient approximation (PAW-GGA) pseudopotentials were adopted to describe the electron-ion interaction. All the structures were optimized aiming to the global energy minimum (Table S6), fully relaxed until the residual force convergence value on each tom being less 0.05 eV/Å. The Brillouin zone was sampled by *3 x 3 x 1* Gamma k-point mesh and the wave functions were expanded using a plane-wave basis set with

kinetic energy cutoff of 500 eV. Spin-polarization was calculations with the lowest energy magnetic configurations were identified. All of the above structures were establised by Materials Studio.

The analysis of free energy is listed as follows.

$$COF\text{-}SO_3H + UO_2(NO_3)_2 \rightarrow COF\text{-}SO_3UO_2NO_3 + HNO_3 \quad (1)$$

$$COF\text{-}SO_3H + NH_3{\cdot}H_2O \rightarrow [NH_4]^+[COF\text{-}SO_3^-]+H_2O \quad (2)$$

$$[NH_4]^+[COF\text{-}SO_3^-]+UO_2(NO_3)_2 \rightarrow COF\text{-}SO_3UO_2NO_3+NH_4NO_3 \quad (3)$$

$$\Delta G_1=E(COF\text{-}SO_3UO_2NO_3)+E(HNO_3)-E(COF\text{-}SO_3H)-E(UO_2(NO_3)_2)$$

$$\Delta G_2=E([NH_4]^+[COF\text{-}SO_3^-])+E(H_2O)-E(COF\text{-}SO_3H)-E(NH_3{\cdot}H_2O)$$

$$\Delta G_3=E(COF\text{-}SO_3UO_2NO_3)+E(NH_4NO_3)-E([NH_4]^+[COF\text{-}SO_3^-])-E(UO_2(NO_3)_2)$$

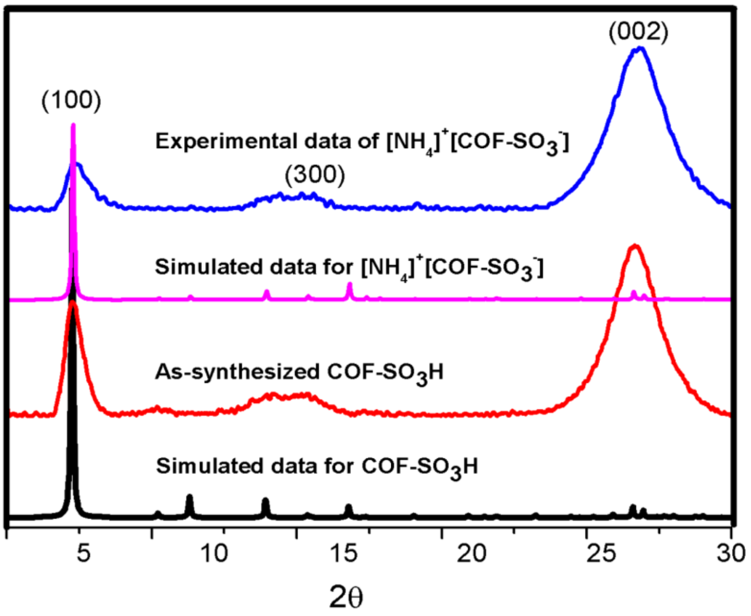


**Figure S1.** The experimental and calculated PXRD patterns of $COF\text{-}SO_3H$ and $[NH_4]^+[COF\text{-}SO_3^-]$.

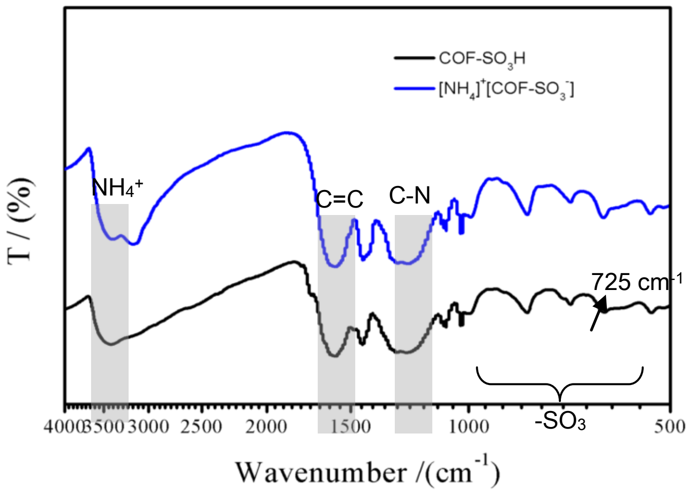


**Figure S2.** The IR bonds of $COF\text{-}SO_3H$ and $[NH_4]^+[COF\text{-}SO_3^-]$.

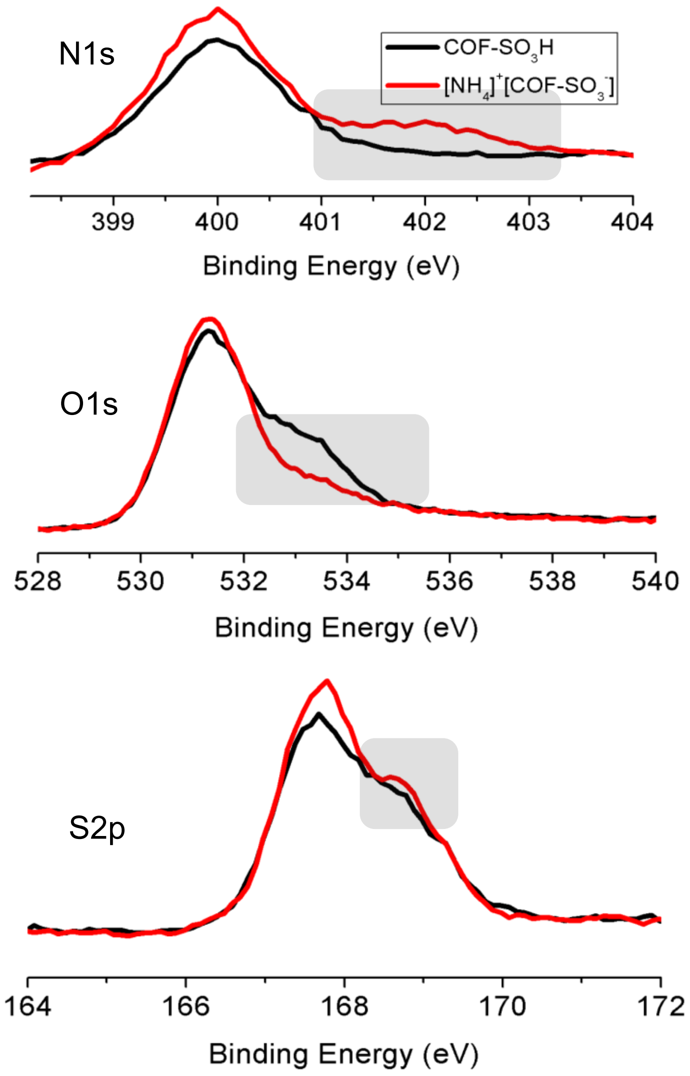


**Figure S3.** The high resolution spectra of N1s, O1s, and S2p of COF-$SO_3H$ and $[NH_4]^+$[COF-$SO_3^-$].

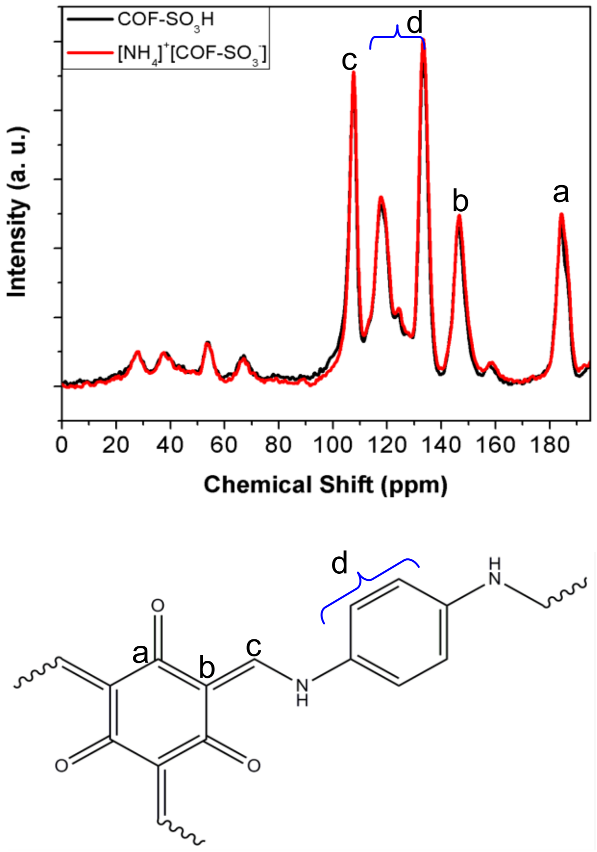


**Figure S4.** $^{13}$C CP-MAS spectrum of COF-$SO_3H$ and $[NH_4]^+$[COF-$SO_3^-$].

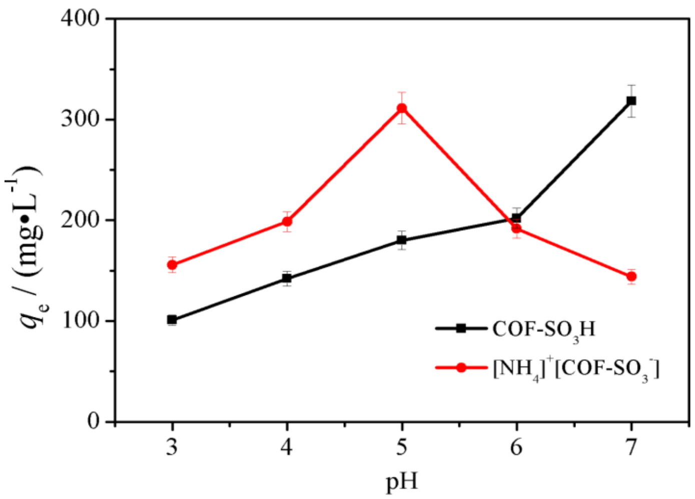


**Figure S5.** The pH-dependent U adsorption on COF-$SO_3H$ and $[NH_4]^+$[COF-$SO_3^-$] materials.

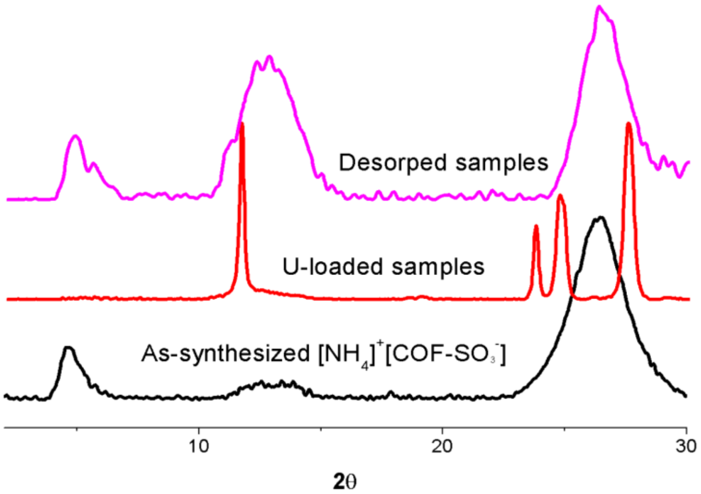


**Figure S6.** A comparison of PXRD patterns among these samples included in as-synthesized $[NH_4]^+[COF\text{-}SO_3^-]$ samples, the samples after U adsorption, and the samples after desorption of U by HCl.

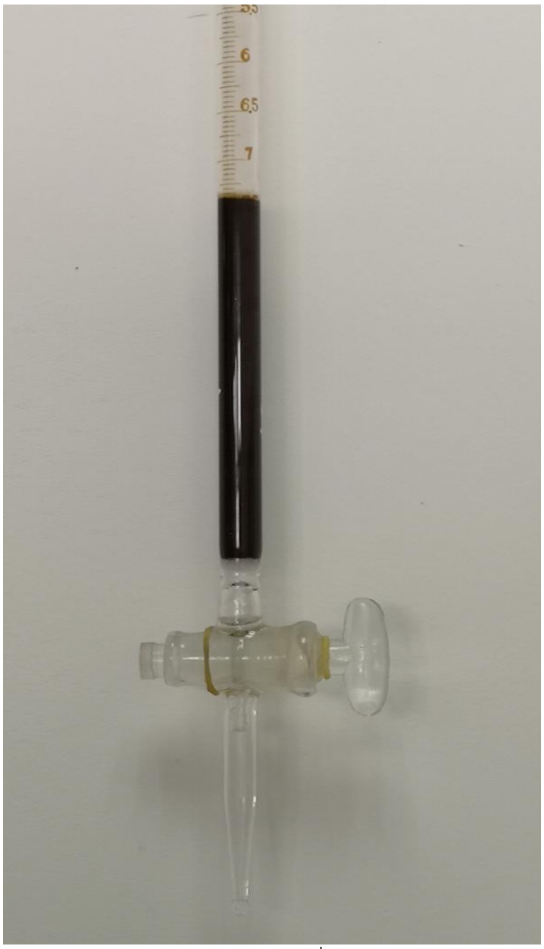

**Figure S7.** The packed bed by means of $[NH_4]^+[COF\text{-}SO_3^-]$ samples used in this work.

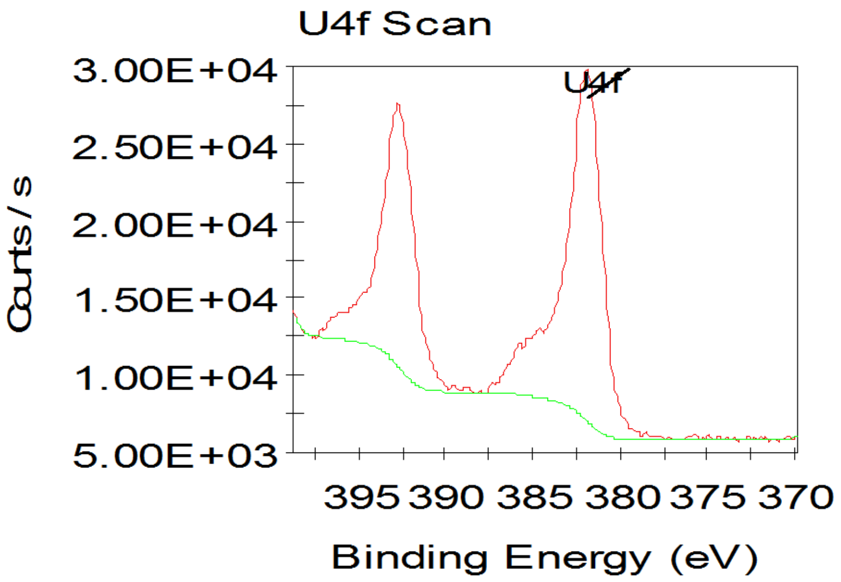


**Figure S8.** The high resolution spectra of $U_{4f}$ for the samples of $[NH_4]^+[COF\text{-}SO_3^-]$ after loading of U.

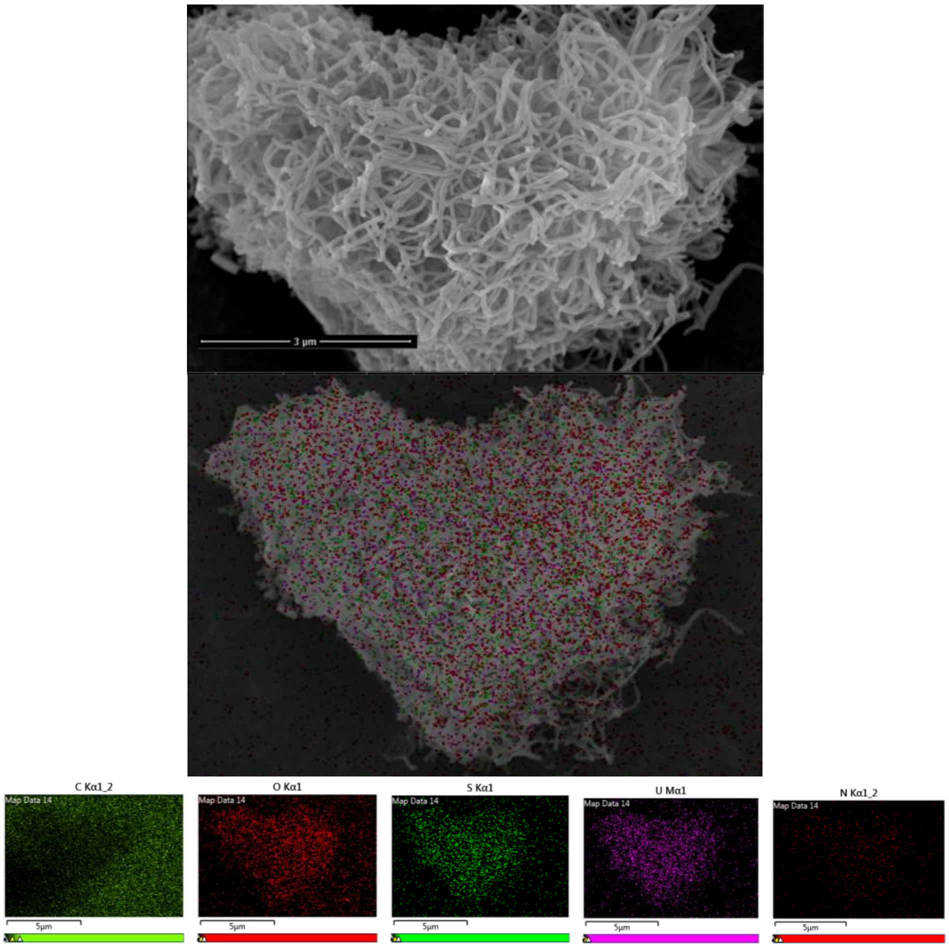


**Figure S9.** SEM and EDS mapping image of the samples of $[NH_4]^+[COF\text{-}SO_3^-]$ after loading of U.

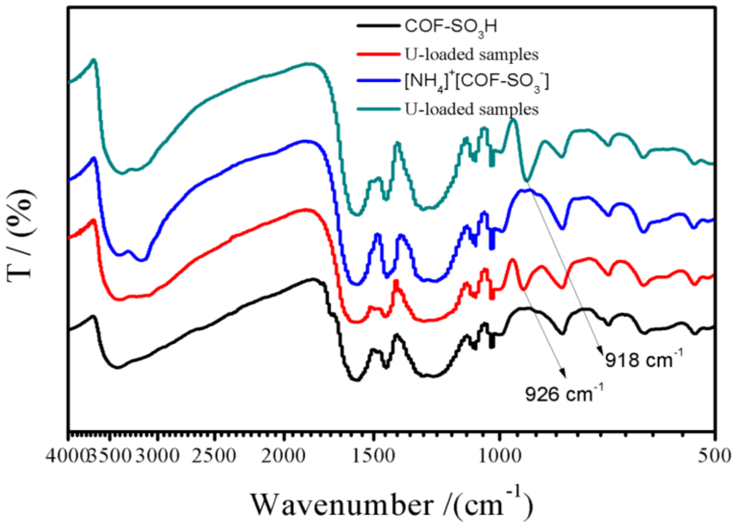


**Figure S10.** The IR bonds of COF-$SO_3$H and $[NH_4]^+$[COF-$SO_3^-$] samples and their corresponding U-loaded samples.

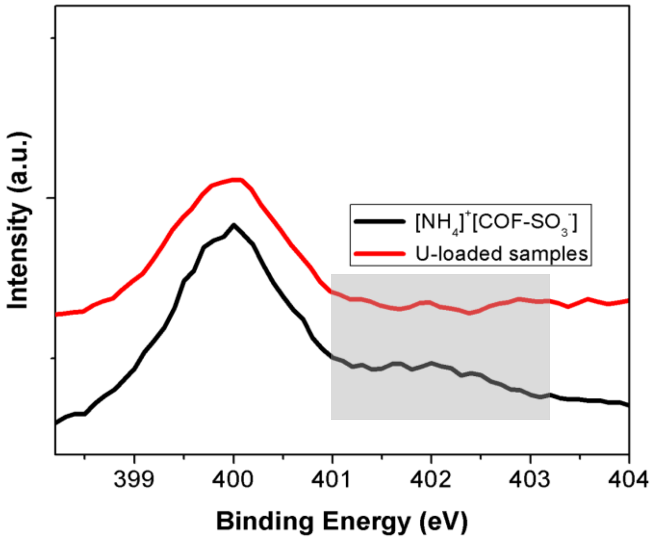


**Figure S11.** A comparison in the high resolution spectra of N1s for $[NH_4]^+$[COF-$SO_3^-$] samples and samples after loading of U.

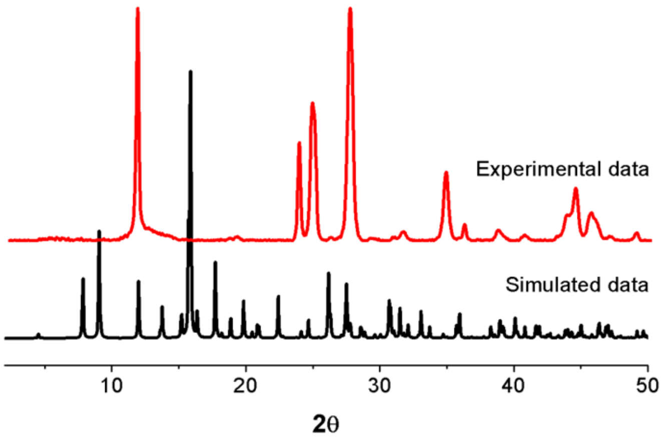


**Figure S12.** The experimental and simulated PXRD patterns for the samples of $[NH_4]^+[COF\text{-}SO_3^-]$ after loading of U.

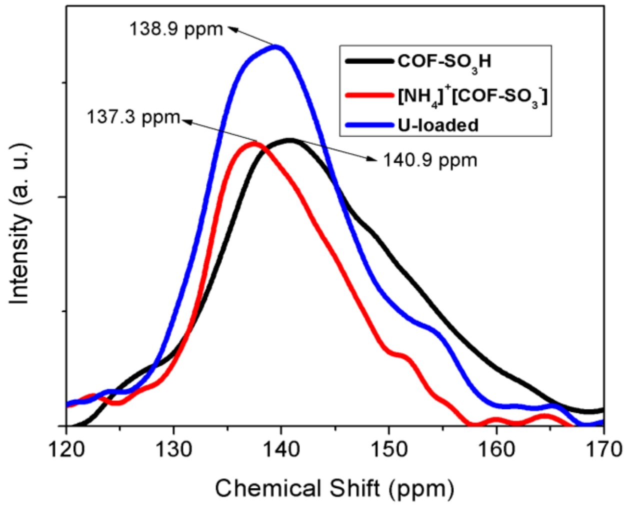


**Figure S13.** A comparison in the $^{15}N$ CP-MAS spectrum of $COF\text{-}SO_3H$, $[NH_4]^+[COF\text{-}SO_3^-]$, and the samples of $[NH_4]^+[COF\text{-}SO_3^-]$ after loading of U.

**Table S1.** Isotherm parameters of Langmuir and Freundlich models of U adsorption by means of $COF\text{-}SO_3H$ and $[NH_4]^+[COF\text{-}SO_3^-]$.

| Adsorbent | Langmuir model | | | Freundlich model | | |
|---|---|---|---|---|---|---|
| | $K_L$/(L·mg$^{-1}$) | $q_m$/(mg·g$^{-1}$) | $R^2$ | $K_F$/(mg$^{1-1/n}$·g$^{-1}$·L$^{-1/n}$) | 1/$n$ | $R^2$ |
| $COF\text{-}SO_3H$ | 0.01322 | 421.941 | 0.9986 | $7.556\times10^3$ | 0.2899 | 0.9632 |
| $[NH_4]^+[COF\text{-}SO_3^-]$ | 0.05551 | 869.565 | 0.9999 | $1.5375\times10^4$ | 0.5721 | 0.8049 |

**Table S2.** A comparison in adsorption capacity among various reported porous adsorbents.

| Adsorbents | Adsorption capacity (mg/g) | Reference |
|---|---|---|
| ARSEN-$X^{np}$ Purolite resin | 47 | Rev. Chim. 2011, 62, 814 |
| AMBERSEP 920U Cl resin | 50 | IOSR J. Appl. Chem. 2014, 7, 32 |
| Tulsion CH-96 | 70 | J. Radioanal. Nucl. Chem. 2008, 275, 563 |
| MSPh-III (phosphonic acid-modified mesoporous material) | 182 | Micropor. Mesopor. Mater.2013,180, 22 |
| Mesoporous Carbon Materials | 97 | Ind. Eng. Chem. Res.2013, 52, 15187 |
| Am-p(AN-c-MAc) | 51.5 | ACS Appl. Mater. Interfaces 2012, 4, 163 |
| MOF-76 | 298 | Chem. Commun.2013, 49, 10415 |
| MIL-101-DETA | 350 | J. Mater. Chem. A 2015, 3, 525 |
| MIL-101(Cr)-triazole-COOH | 304 | ACS Appl. Mater. Interfaces 2016, 8, 31032 |
| $S_x$-LDH | 330 | J. Am. Chem. Soc.2015, 13, 3670 |
| $K_2MnSn_2S_6$(KMS-1) | 380 | J. Am. Chem. Soc.2012, 134, 16441 |
| FJSM-GAS-1 | 196 | J. Am. Chem. Soc. 2018, 140, 11133 |
| FJSM-SnS | 338 | J. Am. Chem. Soc.2016, 138, 12578 |
| COF-TpDb-AO | 408 | Adv. Mater. 2018, 1705479 |
| POP-TpDb-AO | 355 | Adv. Mater. 2018, 1705479 |
| POP-oNH2-AO | 530 | Nat. Commun. 2018, 9:1644 |
| COF-$SO_3$H | 360 | **In this work** |
| $[NH_4]^+$[COF-$SO_3^-$] | 851 | **In this work** |

**Table S3.** Kinetic parameters of pseudo-first-order and pseudo-second-order models of U adsorption by means of COF-$SO_3$H and $[NH_4]^+$[COF-$SO_3^-$].

| Adsorbent | $q_{e,\ exp}$ (mg·g$^{-1}$) | pseudo-first-order | | | pseudo-second-order | | | $K_d$ |
|---|---|---|---|---|---|---|---|---|
| | | $q_{e,cal}$ (mg·g$^{-1}$) | $k_1$ (min$^{-1}$) | $R^2$ | $q_{e,\ cal}$ (mg·g$^{-1}$) | $k_2$ (g·mg$^{-1}$·min$^{-1}$) | $R^2$ | |
| COF-$SO_3$H | 211.429 | 82.836 | 0.1158 | 0.954 | 211.594 | 0.00188 | 0.9999 | $4.228\times10^3$ |
| $[NH_4]^+$[COF-$SO_3^-$] | 383.008 | 143.434 | 0.1078 | 0.962 | 380.517 | 0.00225 | 0.9999 | $5.0835\times10^5$ |

**Table S4.** Kinetic parameters of pseudo-first-order and pseudo-second-order models of U adsorption by means of $[NH_4]^+[COF\text{-}SO_3^-]$ and the samples after γ radiation (5 Gy) for eight days for a 50 ppm U solution.

| Adsorbent | $q_{e,\,exp}$ (mg·g$^{-1}$) | pseudo-first-order | | | pseudo-second-order | | | $K_d$ |
|---|---|---|---|---|---|---|---|---|
| | | $q_{e,cal}$ (mg·g$^{-1}$) | $k_1$ (min$^{-1}$) | $R^2$ | $q_{e,\,cal}$ (mg·g$^{-1}$) | $k_2$ (g·mg$^{-1}$·min$^{-1}$) | $R^2$ | |
| $[NH_4]^+[COF\text{-}SO_3^-]$ | 99.974 | 3.0869 | 0.00682 | 0.6079 | 99.4036 | 0.00724 | 0.9999 | $9.8\times10^6$ |
| After irradiation | 98.796 | 16.3133 | 0.00665 | 0.81935 | 98.1354 | 0.00488 | 0.9999 | $9.1\times10^5$ |

**Table S5.** Kinetic parameters of pseudo-first-order and pseudo-second-order models of U adsorption by means of $[NH_4]^+[COF\text{-}SO_3^-]$ for a 10 ppm U solution.

| pH | $q_{e,\,exp}$ (mg·g$^{-1}$) | pseudo-first-order | | | pseudo-second-order | | | $K_d$ |
|---|---|---|---|---|---|---|---|---|
| | | $q_{e,cal}$ (mg·g$^{-1}$) | $k_1$ (min$^{-1}$) | $R^2$ | $q_{e,\,cal}$ (mg·g$^{-1}$) | $k_2$ (g·mg$^{-1}$·min$^{-1}$) | $R^2$ | |
| pH = 1 | 19.62 | 6.7400 | 0.005204 | 0.72766 | 19.6425 | 0.00794 | 0.9999 | $1.0326\times10^5$ |
| pH = 8 | 18.224 | 13.2068 | 0.00467 | 0.60158 | 18.2815 | 0.01186 | 0.9999 | $2.0522\times10^4$ |
| HCl (3 M) | 12.0258 | 1.5954 | 0.00541 | 0.8317 | 12.0064 | 0.01127 | 0.9997 | $2.2661\times10^3$ |

**Table S6.** The calculated energy for these compounds.

| | $COF\text{-}SO_3H$ | $[NH_4]^+[COF\text{-}SO_3^-]$ | $COF\text{-}SO_3UO_2NO_3$ |
|---|---|---|---|
| E/eV | -1144.16 | -1269.53 | -1455.30 |
